# Differential Gene Expression in Liver Tissues of Streptozotocin-Induced Diabetic Rats in Response to Resveratrol Treatment

**DOI:** 10.1371/journal.pone.0124968

**Published:** 2015-04-23

**Authors:** Gökhan Sadi, Mehmet Cengiz Baloğlu, Mehmet Bilgehan Pektaş

**Affiliations:** 1 Department of Biology, Kamil Ozdag Science Faculty, Karamanoglu Mehmetbey University, Karaman, Turkey; 2 Department of Genetics and Bioengineering, Engineering Faculty, Kastamonu University, Kastamonu, Turkey; 3 Department of Medical Pharmacy, Faculty of Medicine, Afyon Kocatepe University, Afyon, Turkey; Cankiri Karatekin University, TURKEY

## Abstract

This study was conducted to elucidate the genome-wide gene expression profile in streptozotocin induced diabetic rat liver tissues in response to resveratrol treatment and to establish differentially expressed transcription regulation networks with microarray technology. In addition to measure the expression levels of several antioxidant and detoxification genes, real-time quantitative polymerase chain reaction (qRT-PCR) was also used to verify the microarray results. Moreover, gene and protein expressions as well as enzymatic activities of main antioxidant enzymes; superoxide dismutase (SOD-1 and SOD-2) and glutathione S-transferase (GST-Mu) were analyzed. Diabetes altered 273 genes significantly and 90 of which were categorized functionally which suggested that genes in cellular catalytic activities, oxidation-reduction reactions, co-enzyme binding and terpenoid biosynthesis were dominated by up-regulated expression in diabetes. Whereas; genes responsible from cellular carbohydrate metabolism, regulation of transcription, cell signal transduction, calcium independent cell-to-cell adhesion and lipid catabolism were down-regulated. Resveratrol increased the expression of 186 and decreased the expression of 494 genes in control groups. While cellular and extracellular components, positive regulation of biological processes, biological response to stress and biotic stimulants, and immune response genes were up-regulated, genes responsible from proteins present in nucleus and nucleolus were mainly down-regulated. The enzyme assays showed a significant decrease in diabetic SOD-1 and GST-Mu activities. The qRT-PCR and Western-blot results demonstrated that decrease in activity is regulated at gene expression level as both mRNA and protein expressions were also suppressed. Resveratrol treatment normalized the GST activities towards the control values reflecting a post-translational effect. As a conclusion, global gene expression in the liver tissues is affected by streptozotocin induced diabetes in several specific pathways. The present data suggest the presence of several processes which contribute and possibly interact to impair liver functions in type 1 diabetes, several of which are potentially amenable to therapeutic interventions with resveratrol.

## Introduction

Diabetes mellitus is one of the most severe endocrine metabolic disorders associated with the absence or decreased level of insulin hormone or its reduced action on cells. It induces serious complications including coronary artery, renal and ophthalmologic diseases resulted in disability or mortality of diabetic patients [1]. Microarray technique provides possibility to compare the gene expressions of different cells or tissue types in a wide scale. It has important usage areas such as; revelation of the subgroups of certain diseases, development of new detection methods and understanding the molecular mechanism of a response that is generated against a disease or drug.

Recently, expression microarrays have been widely used to characterize gene expression patterns in diabetes. For instance, 46 genes were found to be altered in lung tissues of diabetic rats. Among these; apoptosis, response to stress, regulation of protein kinase activity, ion transporter activity and collagen synthesis were dominated [2]. Moreover, diabetes modulated 97 and 102 genes of diaphragm and sternohyoid muscle of Zucker diabetic fatty rats, respectively. The genes responsible from lipid and carbohydrate metabolisms, muscle contraction, ion transport and collagen metabolism were changed considerably and a shift in gene expression from carbohydrate metabolism toward lipid metabolism were identified [3]. After 24 hours from streptozotocin (STZ) treatment, expressions of more than 100 genes were altered and this number declined in a time dependent manner in mouse liver tissues. Expression of stress/xenobiotic metabolism, cell cycle and apoptosis-related genes increased, while genes which are associated with glucose, lipid and protein metabolisms decreased after STZ treatment [4]. Gene expression profile by high-throughput sequencing in type 2 diabetic mouse livers were also studied, and 2627 genes were found to be modulated significantly. Gene ontology analysis showed that the up-regulated genes were mainly enriched in metabolism whereas down-regulated genes were mainly enriched in immune-related processes [5].

Resveratrol (3,4’,5-trihydroxystilbene) is a polyphenolic compound which is produced by plants against various infections. The cardio protective, anti-cancer and anti-inflammatory actions were investigated with various studies [6–8]. Resveratrol has become increasingly attractive as a therapeutic agent in the treatment of a variety of pathologies, including diabetes mellitus [9]. Although its antioxidant activity has been demonstrated previously [8,10,11], the mechanisms underlying the beneficial effects of resveratrol have not been completely elucidated. Recently, it has been found that resveratrol may act on some transcription factors such as FoxO-1, Nrf2 and NFκB to increase the expression of several genes so that it can normalize the changes which are caused from diabetes [12,13].

Resveratrol affected expression of more than 1200 genes in breast cancer cell line, MCF-7. These genes involved in mismatch repair, DNA replication, homologous recombination and cell cycle [14]. In another current study, microarray analysis revealed that resveratrol down-regulated Wnt and Notch signaling pathways and up-regulated the genes of cell cycle regulation in adipocytes [15]. In a study over turbot head kidney leucocytes, resveratrol repressed expression of several genes which are involved in immune responses and inflammation, and induced several cytoskeleton-related genes [16]. Resveratrol also altered the expression of more than 1600 transcripts in human prostate cancer cells, mostly belonging to androgen pathway, in a time and dose dependent manner [17].

Even though these recent advances reveals *in vitro* effects of resveratrol on gene expression profile of some cell lines, there is still a lack of knowledge about the changes in gene expression profiles in animal models of diabetes in response to resveratrol. To understand tissue-specific molecular alterations in diabetes and *in vivo* effects of resveratrol; we hypothesized that diabetes-related alterations in global gene expression profile of liver tissues could be casually returned to normal values by resveratrol. To make track for the molecular action mechanism of the resveratrol through the regulation of the liver function, the present study was designed to examine the gene expression profile in liver tissues of rats treated with resveratrol using microarray technology.

## Materials and Methods

### Materials

Streptozotocin and pyrogallol were purchased from Sigma (St. Louis, MO, USA); trans-resveratrol was obtained from Molekula (Gillingham, Dorset, UK). Total RNA isolation kits and reagents for cDNA synthesis were obtained from Thermo Scientific (Burlington, Canada). SYBR Green I Master Mix was purchased from Roche (Foster City, CA, USA). GeneChip Rat Gene 2.0 ST Arrays, IVT express kits, GeneChip hybridization, wash and stain kits were obtained from Affymetrix (Santa Clara, USA). Antibodies were obtained from Abcam (Cambridge, MA, USA) and Santa Cruz (Santa Cruz, CA, USA). PVDF membranes were purchased from Bio-Rad (Hercules, California, USA). All other chemicals used in this study were of the highest analytical grade available, and the buffers were prepared using sterile distilled water.

### Animal Procedure and Tissue Preparation

Experiments were performed on eight-week-old adult male Wistar rats weighing between 300–350 g. Study protocols were approved in advance by the local ethics committee for animal research studies at the Karamanoglu Mehmetbey University (K.M.U. ET-11/01-02). This study was carried out strictly according to rules of the guide for the care and use of laboratory animals as published by the US National Institute of Health (NIH Publication No: 85/23, revised in 1986). All efforts were made to minimize animal suffering. A completed ARRIVE guideline [18] is included in S1 File.

Rats were housed under temperature-controlled rooms (20–22°C) with a 12-hours light-dark cycle. The animals were fed with standard rodent diet composed of 62% starch, 23% protein, 4% fat, 7% cellulose, standard vitamins and salt mixture (chow pellet). After acclimation for one week, animals were randomly assigned into four groups: (1) non-diabetic control group (n = 12) given only vehicle (10% DMSO), (2) non-diabetic control group (n = 12) given a daily i.p. dose of 20 mg/kg/day resveratrol in 10% DMSO (K+RSV) throughout the 4-week period, (3) diabetic group (n = 12) received STZ (55 mg/kg) dissolved in citrate buffer (0.05 M, pH: 4.5) and vehicle for each day and (4) diabetic group treated with resveratrol (n = 9) (D+RSV), which received a daily i.p. dose of 20 mg/kg/day resveratrol throughout the 4-week period, starting from two days after STZ administration. Blood glucose concentrations were determined by Accu-check-go glucometer (Roche, Germany) weekly from blood of tail veins. The criteria for the diabetes were the blood glucose concentration higher than 200 mg/dl. After 4 weeks of diabetes, the rats were fasted overnight and decapitated for the removal of liver tissues, which were quickly frozen in liquid nitrogen and kept at -85°C for subsequent biochemical analyses.

### Total RNA Isolation and Microarray Analysis

Total RNAs were isolated from liver tissues using the RNeasy total RNA isolation kit (Qiagen, Venlo, Netherlands) according to the manufacturer protocol. After isolation, the amount and the quality of total RNA were determined using spectrophotometry at 260/280 nm and the Agilent 2100 bioanalyzer (Santa Clara, USA). Samples, whose RNA integrity number (RIN) greater than 7.0 were used in qRT-PCR experiments and the samples having the highest RIN numbers (minimum 8.0) were used in microarray analysis.

First-strand cDNA synthesis was achieved using 0.5 μg total RNA with T7 oligo (dT) primers. Then, single-stranded cDNA was converted into second-strand cDNA which was used as a template for transcription reaction. Amplified RNA (aRNA) was produced from the double-stranded cDNA via *in vitro* transcription (IVT). aRNA was labeled with biotin-conjugated nucleotides in IVT labeling reaction. 15 μg of biotin-labeled aRNA was fragmented and hybridized to Affymetrix rat genome 230 2.0 arrays which represents 31,000 transcripts and provides comprehensive coverage of the entire transcribed rat genome. The hybridized arrays were scanned using an Affymetrix scanner. GeneChip Operating Software 1.4 was used for the extraction of microarray data from scanned GeneChip images. Four biological replicates from four treatment groups were arrayed to rat genome 230 2.0 arrays, totaling 4 arrays × 4 groups.

Analysis of microarray data from all hybridizations and array normalization were done using GeneSpringGX 11.0 (Agilent, USA) software. The raw data were normalized per chip using Robust Multiarray Analysis (RMA) and significantly modulated transcripts were determined with a fold change cut-off threshold of two-fold up- and down-regulation with One-way ANOVA (p<0.05). Microarray data were deposited to ArrayExpress database (http://www.ebi.ac.uk/arrayexpress) under accession number of E-MTAB-3293. According to Principle Component Analysis (PCA) of array data, consistency of biological replicates and differences between four different experimental groups were verified. Gene Ontology Enrichment Analysis Software Toolkit (GOEAST) is used to match significantly changed probe sets with gene ontology groups [19]. Pathway analysis were carried out with web based KEGG (Kyoto Encyclopedia of Genes and Genomes) software [20].

### Quantitative Real Time PCR (qRT-PCR)

The reliability of microarray data was tested by qRT-PCR analysis of the selected genes including *CAT*, *Usp2*, *Igfbp2*, *GST-A5*, *CYP8B1*, and *CYP1A1*. Primer sequences were designed according to appropriate T_m_, %GC and ΔG values by primer express 2.0 software and synthesized by Iontek (Bursa, Turkey). Each primer was homology searched by NCBI BLAST to ensure that they were specific for the target mRNA transcript [21]. In addition to validation experiments, expression levels of various antioxidant (*SOD-1*, *SOD-2*, *GST-Mu*, and *GST-Pi*) and detoxification genes (*CYP1A1*, *CYP1A2*, *CYP2A1*, *CYP2B1*, *CYP2E1*, *CYP2C11*, *CYP4A1*, *CYP8B1*) were also determined.

To do this, 1 μg of total RNA were reverse transcribed to cDNA using commercial first strand cDNA synthesis kit (Thermo Scientific, USA) and gene expressions were determined by mixing 1 μl cDNA, 5 μl 2X SYBR Green Master mix (Roche FastStart Universal SYBR Green Master Mix) and primer pairs (S1 Table) at 0.5 mM concentrations in a final volume of 10 μl. Next, qRT-PCR (LightCycler480 II, Roche, Basel, Switzerland) was performed as follows: initial denaturation at 95°C for 10 minutes, denaturation at 95°C for 10 seconds, annealing at 58°C for 15 seconds and extension at 72°C for 15 seconds with 45 repeated thermal cycles measuring the green fluorescence at the end of each extension step. The PCR reactions were performed in triplicates and the specificity of PCR products were confirmed using melt analysis. The relative expression of genes with respect to internal control; glyceraldehyde 3-phosphate dehydrogenase (*GAPDH*) were calculated with the efficiency corrected advance relative quantification tool provided by the LightCycler 480 SW 1.5.1 software.

### Tissue Homogenization and Protein Extraction

Homogenates of liver tissues were obtained with the aid of Tissue Rupture (Qiagen, Venlo, Netherland) homogenizer using a homogenization solution consisting of 1.15% (w/v) KCl, 5 mM EDTA, 0.2 mM PMSF, and 0.2 mM DTT in 25 mM phosphate buffer at pH: 7.4. Next, homogenates were centrifuged at 1500 *g* for 10 min at 4°C and the supernatants were aliquoted to perform enzyme assays and other biochemical analysis. Protein concentrations were determined according to the Lowry method [22]. Hepatic insulin concentrations were measured using commercially available specific Rat ELISA kits (DRG Instruments GmbH, Germany) according to the manufacturer's protocol.

### Determination of Tissue Oxidant and Antioxidant Status

Measurement of total antioxidant status (TAS) and total oxidant status (TOS) was performed using a total antioxidant and oxidant status determination kits (Rel Assay Diagnostic, Turkey) as described according to the manufacturer protocol. Measurements were carried out by ChemWell 2910 Elisa plate reader (Awareness Technology, Inc. Martin Hwy. Palm City, USA) and results were given as μmol Trolox Equiv./g protein or μmol H_2_O_2_ Equiv./g protein, respectively.

Reduced and oxidized glutathione (GSH and GSSH) levels were determined by HPLC chromatography having a fluorescent detector (Ex: 385 Em: 515 nm) using commercial kits (Chromsystems Diagnostics, Munich, Germany) according to user manual. Results were given as μmol/g protein.

Malonedialdehyde (MDA) levels were determined by HPLC chromatography having a fluorescent detector (Ex:515 Em:553 nm) using commercial kits (Chromsystems Diagnostics, Munich, Germany). Results were given as μmol/g protein.

### Sodium Dodecyl Sulfate (SDS)-Polyacrylamide Gel Electrophoresis (PAGE) and Immunoblot Analysis

For the determination of SOD-1, SOD-2 and GST-Mu protein contents, whole homogenates containing 10 μg (20 μg for GST-Mu) of proteins were separated by SDS-PAGE and electroblotted onto PVDF membranes [23]. Blotted membranes were then blocked with 5% (w/v) bovine serum albumin and incubated with prevalidated [24,25] primary antibodies; SOD-1 (Anti-SOD-1 Sheep IgG, Calbiochem- 574597, Billerica, USA, 1:5000), SOD-2 (Anti-SOD-2 Rabbit IgG, Santa Cruz- sc-30080, USA, 1:100), GST-Mu (Anti-GST-Mu Rabbit IgG, Abcam- ab77925, Cambridge, USA, 1:6000) for two hours. As an internal control, GAPDH proteins were also labeled with anti-GAPDH Rabbit IgG (Santa Cruz-sc-25778, USA, 1:2000) for the normalization. Horseradish peroxidase (HRP) conjugated secondary antibodies (Goat Anti-rabbit-(or sheep for SOD-1) IgG-HRP conjugate; sc-2030 & sc-2770, Santa Cruz, USA, 1:10,000) was incubated for 1 h and the blots were treated with Clarity Western ECL (Bio-Rad Laboratories, Hercules CA, USA) substrate solution. Images of the blots were obtained using the ChemiDoc MP Chemiluminescence detection system (Bio-Rad Laboratories, Hercules CA, USA) equipped with a CCD camera. The relative expression of proteins with respect to GAPDH was calculated using the ImageLab4.1 software.

### Determination of Enzymatic Activities

Total SOD activities were measured by following the inhibition of pyrogallol autoxidation spectrophotometrically [26]. KCN (1.5 mM) were included to assay medium to measure only SOD-2 activity. SOD-1 activities were found by subtracting SOD-2 activities from total SOD activities. One unit of SOD activity was calculated as the amount of protein causing 50% inhibition of pyrogallol autoxidation. GST activities were monitored by the increase in the absorbance of CDNB-GSH (for total GST) or DCNB-GSH (for GST-Mu) adduct as described elsewhere [27].

### Statistical Analysis

Protein expression data were normalized to the mean of the control groups, which was arbitrarily set to 1-fold, and the relative changes were given as fold changes over control. Other data were presented as mean ± standard error of the mean. SPSS 15.0 statistical software (IBM Corporation, Armonk, NY, USA) was used to calculate the statistical significance between groups, which was determined using one-way ANOVA with the appropriate post-hoc test (Tukey’s Honestly Significant Difference). A probability of 0.05 was established as the level of significance in the data analysis.

## Results

### Effect of Resveratrol on STZ Induced Hyperglycemia and Body Weight

Recently, we have publicized that STZ treated rats displayed a significant induction in fasting blood glucose concentration compared to age-matched controls and resveratrol did not affect the diabetic blood glucose levels significantly [13]. In diabetes, albeit blood glucose concentrations were very high, diabetic tissues could not make use of blood glucose and it is possible to observe a shift in energy metabolism towards the usage of lipids and proteins. This might explain the weight loss (Table 1) observed in diabetic animals. Furthermore, resveratrol has been shown to possess calorie restriction effects in animals [28,29], a possible explanation of slight decrease in body weight observed with resveratrol treatment.

**Table 1:** Summary of overall changes in animals’ weights and tissue oxidant /antioxidant status markers.

	Control	Diabetes	K+RSV	D+RSV
Initial body weight (g)	440.81 ± 21.03	393.74 ± 11.23	399.56 ± 6.96	392.61 ± 6.94
Final body weight (g)	447.70 ± 16.93	319.30 ± 16.86 *	411.82 ± 14.41 #	309.43 ± 14.62 *
Initial blood glucose (mg/dl)	101.60 ± 3.40	98.80 ± 7.90	96.90 ± 8.10	103.90±8.60
Blood glucose (mg/dl)after STZ treatment	102.50 ± 6.80	401.70 ± 27.60 *	107.30 ± 10.23 #	373.30 ± 34.20*
Hepatic Insulin(μg/g protein)	134.07 ± 15.71	46.80 ± 6.95 *	128.18 ± 21.09 #	54.43 ± 6.05
TASμmol Trolox Equiv./g protein	0.19 ± 0.02	0.18 ± 0.01	0.34 ± 0.05 * #	0.24 ± 0.01
TOSμmol H2O2 Equiv./g protein	31.87 ± 4.51	54.57 ± 3.67 *	26.66 ± 1.75 #	39.20 ± 1.70
MDAμmol/g protein	0.065 ± 0.014	0.116 ± 0.015 *	0.074 ± 0.009 #	0.069 ± 0.006 #
GSHμmol/g protein	43.49 ± 4.85	87.28 ± 3.32 *	28.97 ± 2.31 #	85.34 ± 3.88 *
GSSHμmol/g protein	14.39 ± 0.96	45.42 ± 1.15 *	8.86 ± 1.02	39.08 ± 2.90 *
GSH/GSSH	2.98 ± 0.21	1.91 ± 0.08 *	3.38 ± 0.19 #	2.22 ± 0.11

Data were expressed as mean ± Standard Error of Mean (S.E.M)

*represents significance at p<0.05 as compared with control groups.

^#^represents significance at p<0.05 as compared with untreated diabetic groups.

### Effect of Resveratrol on Oxidant and Antioxidant Status in Diabetic Liver

In our previous study, statistically significant elevation in protein carbonylation and decrease in protein thiols were found in diabetic livers, suggesting diabetes-promoted oxidative stress [13]. In this study, presence of oxidative stress in liver tissues was also evidenced with the increment in TOS and MDA levels and decrease in GSH/GSSH ratio by STZ administration.

Resveratrol significantly altered TAS and GSH/GSSH ratios as given to the control rats suggesting that it has strong antioxidant properties to increase the antioxidant potential in the tissues. It normalized elevated TOS and MDA levels and decreased GSH/GSSH ratio observed in diabetic group. Table 1 summarizes the results obtained from the biochemical analysis of oxidative biomarkers with both STZ and resveratrol treatment.

### Microarray Results

After array hybridization and deep scanning, data were normalized with RMA method and significantly altered probe sets were determined whose expression levels were at least 2-fold modulated. Table 2 summarizes the total number of up- and down-regulated genes with STZ or/and resveratrol treatment. Microarray data are available in the ArrayExpress database (http://www.ebi.ac.uk/arrayexpress) under accession number of E-MTAB-3293[30].

**Table 2:** Summary of up- and down-regulated probe set numbers.

*Comparison*	*Up-regulatedprobe set number*	*Down-regulatedprobe set number*
Control vs. Diabetes	90	183
Control vs. K+RSV	186	494
Control vs. D+RSV	214	310
Diabetes vs. D+RSV	159	97
D +RSV vs. K+RSV	393	218

Total numbers of probe sets having at least 2-fold altered expression as compared to reference group that is given initially in the first column. K+RSV: Resveratrol supplemented control group; D+RSV: Resveratrol supplemented diabetic group.

**Fig 1:**
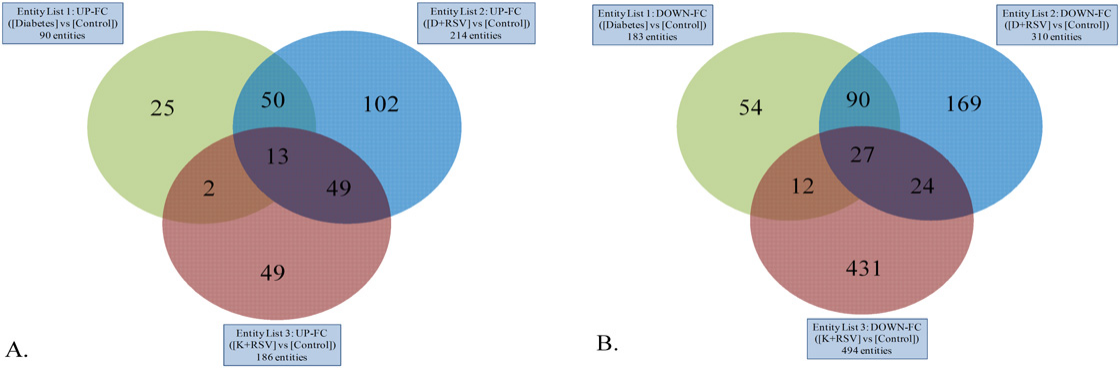
Venn diagrams. Differentially up-regulated (a) and down-regulated (b) probe set numbers in diabetic, D+RSV and K+RSV groups as compared to control group. The number of genes unique to each group is shown inside the circle, the number of genes changed in two groups is shown in the shaded overlap—ping areas, and the number of genes whose expression profile was modified in all three groups is shown in the central overlap. K+RSV: Resveratrol supplemented control group; D+RSV: Resveratrol supplemented diabetic group.

As compared to the control group, STZ and resveratrol up-regulated the expression of 90 and 186 transcripts, respectively. As given together, 214 genes had enhanced expression. Moreover, diabetes down-regulated 183, and resveratrol down-regulated 494 genes. Diabetes and resveratrol together decreased the expression of 310 transcripts. Total number of genes, whose expression levels were significantly modulated in experimental groups, were summarized in Venn diagrams (Fig 1).

Accordingly, some of the genes were found to be effected from diabetes and resveratrol or both in the same manner. That is, shared 13 genes were up-regulated in all three; diabetic, K+RSV and D+RSV groups. Meanwhile, 27 genes were down-regulated in all groups as compared to controls.

In both diabetic and D+RSV group, the same 63 transcripts were repressed while the same 117 genes had increased expression levels. Comparing the effects of resveratrol and diabetes on the gene expression pattern separately, both treatment enhanced same 62, and reduced same 51 transcript levels. As given to the diabetic animals, resveratrol further enhanced 159 and reduced 97 genes significantly (p<0.05). K+RSV group had 476 highly expressed genes and 477 low expressed genes as compared to untreated diabetic rats. According to another Venn diagram analysis (data not shown), as resveratrol were given to diabetic and control groups, 97 genes (up-regulated 78, down-regulated 19 genes) behaved similarly.

Significantly modulated probe sets were analyzed for their gene ontology (GO) with web based software GOEST [19] and their identities were checked from NCBI with Entrez gene numbers.

### Effects of STZ on Liver Gene Expression Profile

**Fig 2:**
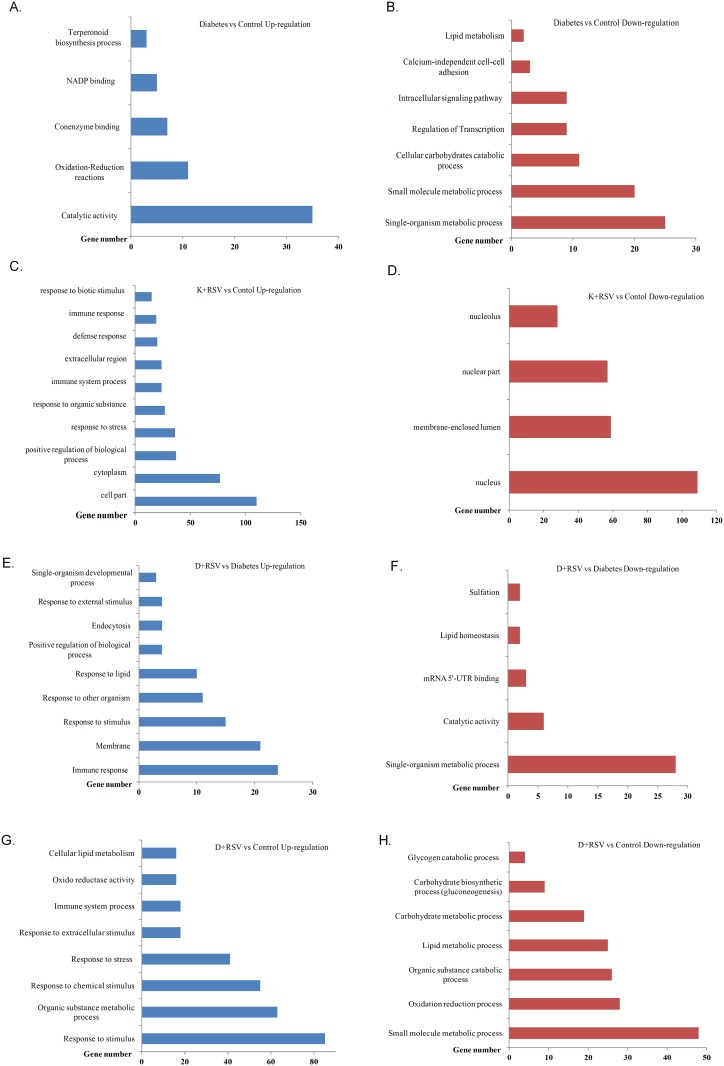
Functional classification of differentially expressed genes with STZ and resveratrol treatments. Numbers of up-regulated (A, C, E, G) and down-regulated (B, D, F, H) genes with diabetes and resveratrol are summarized. One gene could be classified under more than one biological process. K+RSV: Resveratrol supplemented control group. D+RSV: Resveratrol supplemented diabetic group.

The up-regulated 90 genes were functionally annotated into five distinct groups in such a way that genes responsible from cellular catalytic activities, oxidation-reduction reactions, coenzyme binding, NADP binding and terpenoid biosynthesis had at least two fold increased expression. The down-regulated 183 genes were responsible for carbohydrate metabolism, transcriptional regulation, cellular signal transduction, calcium independent cell-to-cell adhesion and lipid catabolism with at least 2-fold reduced expression. The number of STZ-induced genes whose ontological classification was carried out is summarized in Fig 2A and 2B. Whole list of the genes, whose expression levels were significantly up- and down-regulated with STZ treatment (diabetes), ontological groups, probe numbers, fold change values as well as gene symbols were given in supporting information file (S2 Table) to this manuscript.

### Effects of Resveratrol on Liver Gene Expression Profile

Resveratrol, which is a strong antioxidant and regulatory compound, increased the expression of 186 genes that were categorized into ontological groups. GO analysis demonstrated that genes that are in charge from cytoplasmic, cellular and extracellular components were up-regulated. In addition, expression of genes related with positive regulation of biological processes, responses to stress stimulus, responses to biotic factors and immune reactions increased significantly (Fig 2C). In the K+RSV group, 494 genes were significantly down-regulated and differentially regulated genes in this group were mostly nucleus and nucleolus related. Resveratrol treatment resulted in physiological inhibition of genes responsible from functional proteins present in nuclear compartments such as nucleolus, nuclear envelop and nuclear lumen (Fig 2D).

### Effects of Resveratrol on Diabetic Rat Liver Gene Expression Profile

As given to the diabetic animals, resveratrol modulated the expression of 256 genes (159 up-regulated, 97 down-regulated) significantly with at least 2-fold alteration. Induced genes were categorized into groups in such a way that genes functioning in immune response, internal and external stimuli, positive regulation of biological process, membrane constituents and developmental processes were up-regulated (Fig 2E). D+RSV group had also down-regulated genes as compared to diabetic group. These genes involved in single cell metabolic processes, catabolic reactions, lipid homeostasis, sulfation and mRNA processing (Fig 2F). List of these genes with symbols, entrez gene numbers and fold change values were also given in S2 Table.

### Effects of Both STZ and Resveratrol Administration Simultaneously on Rat Liver Gene Expression

In concert with diabetes, resveratrol induced 214 and repressed 310 genes as compared to untreated control group. According to GO analysis, up-regulated genes belonged mostly to responses to stimuli (stress, extracellular factors and chemicals). In addition, genes responsible from cellular lipid metabolism, oxidoreductase activity, immune system processes and metabolic responses to organic compounds were induced (Fig 2G). D+RSV group had also at least 2-fold significantly down-regulated genes most of which involved in glycogen catabolism, biosynthetic processes of carbohydrates (gluconeogenesis), lipid and carbohydrate metabolism, catabolism of organic compounds, oxidation reduction reactions and metabolic processes of small molecules (Fig 2H).

There were number of genes whose expression profile was modified similarly in all three groups (Diabetes, D+RSV and K+RSV). These three groups had 13 up-regulated and 27 down-regulated genes whose expression levels were above or below the control values, respectively. Among these common genes, there was not any strong association to put them into specific GO term. According to data, *Usp2* was the most up-regulated ones while *Nrep* was the most down-regulated ones. It is interesting to mention that fold change values of up- or down-regulated genes in STZ or resveratrol groups were boosted in a cumulative pattern as given together. For instance, *Usp2* levels were 15.97 fold up-regulated in diabetics and 7.41 fold in resveratrol groups. As STZ and resveratrol were given together to same group (D+RSV), up-regulation levels become 16.27 fold. Similarly, *Nrep* mRNAs were 3.40 fold down-regulated with diabetes and 3.12 fold down-regulated with resveratrol, but D+RSV group have 27.25 fold decreased expression levels.

### Pathway Analysis

**Fig 3:**
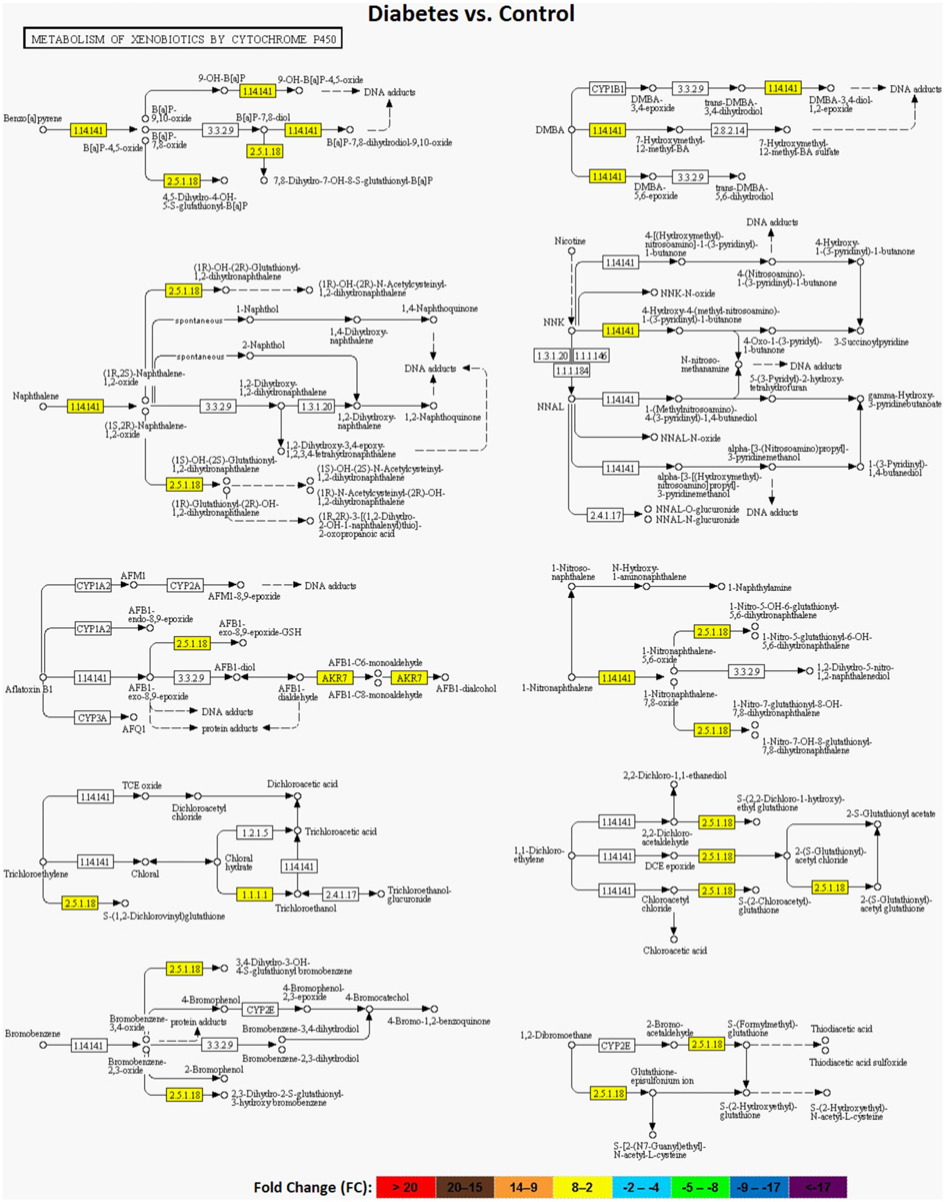
Regulation of cytochrome P450 mediated xenobiotic metabolism with diabetes. Yellow color in the pathway components indicates a general up-regulation of xenobiotic metabolism with diabetes.

KEGG pathway analysis was performed to further elucidate the modulated pathways in STZ and resveratrol treated rat liver tissues [20]. The top KEGG pathways with associated genes and symbols, which are significantly correlated with diabetes and resveratrol, were shown in S1 Fig. Pathway analysis confirmed the modulation of xenobiotic and carcinogen metabolisms with both STZ (Fig 3) and resveratrol. Retinol metabolism and insulin signaling pathways were also modulated in diabetic rat liver tissues. Besides, main biological processes altered by resveratrol were analyzed in cell cycle and cancer, PI3K-AKT, chymokine and MAPK signaling pathways. It could be inferred from the data that down-regulated genes were enriched in programmed cell death cascades and pathways in cancer, suggesting that effects of resveratrol is correlated with apoptosis and cancer. Resveratrol also modulated the cytochrome P450 mediated xenobiotic metabolism, steroid hormone biosynthesis proteins, retinol and NFκB signaling pathways as administered to diabetic animals.

### Validation of Microarray Data by Real Time qRT-PCR

**Fig 4:**
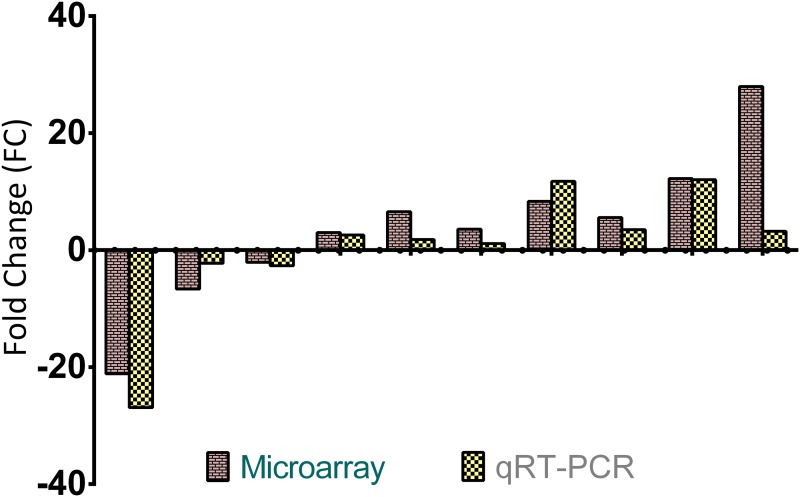
Validation of the microarray data with qRT-PCR. The expressions of six genes of interest between different groups were analyzed using Affymetrix microarray and qRT-PCR. Expression levels of selected genes showed similar directional changes. Since one gene is used for multiple comparisons between different groups, the number of bars in the figure exceeds the total number of genes used in validation study.

To validate microarray data, the expression patterns of several genes of interest (e.g. *CAT*, *Usp2*, *Igfbp2*, *GST-A5*, *CYP8B1 and CYP1A1*) were determined by qRT-PCR analysis. The changes in the expression of the genes were similar in the direction and the magnitude between the two techniques (Fig 4).

### Determination of Gene Expression Levels of Antioxidant and Detoxification Enzymes

In this study, gene expression of main antioxidant enzymes; superoxide dismutases (*SOD-1* and *SOD-2*) and several phase I and phase II detoxification enzymes such as; cytochrome P450s (*CYP1A1*, *CYP1A2*, *CYP2A1*, *CYP2B1*, *CYP2E1*, *CYP 2C11*, *CYP4A1*, *CYP8B1*) and glutathione S-transferases (*GST-Mu* and *GST-Pi*) were determined by qRT-PCR for deep scanning of exact modulation mechanisms over those enzymes.

**Fig 5:**
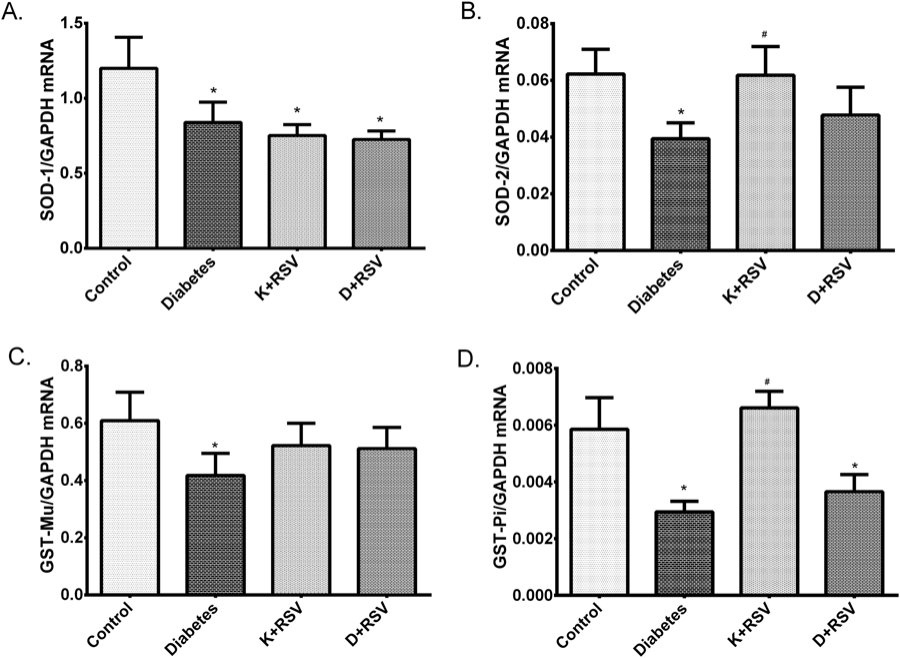
Relative gene expression levels of *SOD-1* (A), *SOD-2* (B), *GST-Mu* (C) and *GST-Pi* (D). Data were normalized with respect to internal standard, *GAPDH*. *Indicates that the means were significantly different (p<0.05) compared to control groups, #Indicates that the means were significantly different (p<0.05) compared to diabetic groups. K+RSV: Resveratrol supplemented control group, D+RSV: Resveratrol supplemented diabetic group.

Quantitative real time PCR results demonstrated that gene expression levels of main antioxidant enzymes *SOD-1*, *SOD-2* and detoxification enzymes *GST-Mu* and *GST-Pi* were suppressed with diabetes (Fig 5). Resveratrol down-regulated *SOD-1* in control group and slightly normalized the *SOD-2* and *GST-Mu* gene expression in diabetic rat liver tissues.

**Fig 6:**
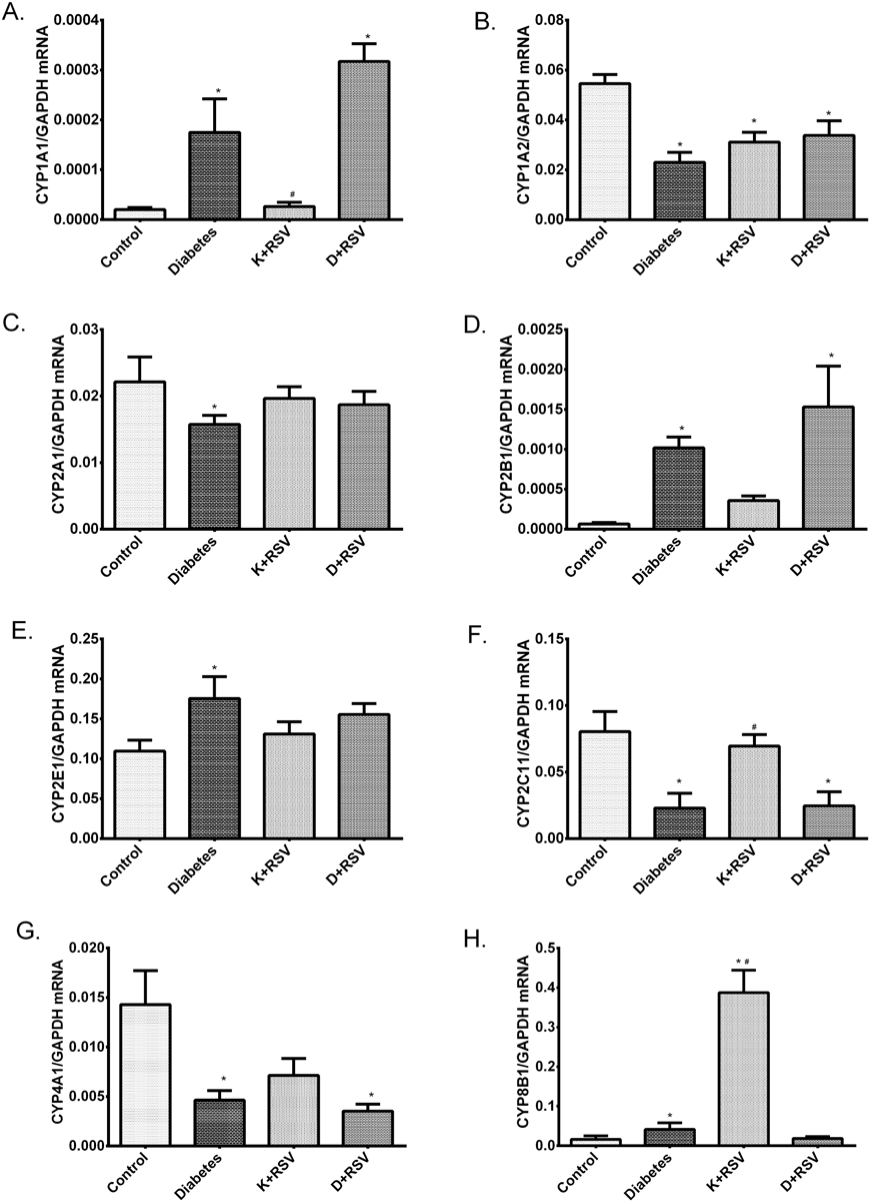
Gene expression levels of cytochrome P450 isozymes with diabetes and resveratrol. Relative gene expression levels of *CYP1A1* (A), *CYP1A2* (B), *CYP2A1* (C), *CYP2B1* (D), *CYP2E1* (E), *CYP2C11* (F), *CYP4A1* (G) and *CYP8B1* (H) with respect to internal standard *GAPDH*. *Indicates that the means were significantly different (p<0.05) compared to control groups, #Indicates that the means were significantly different (p<0.05) compared to diabetic groups. K+RSV: Resveratrol supplemented control group, D+RSV: Resveratrol supplemented diabetic group.

It was also demonstrated that STZ up-regulated *CYP1A1*, *CYP2B1*, *CYP2E1*, *CYP8B1* and down-regulated *CYP1A2*, *CYP2A1*, *CYP2C11*, *CYP4A1* genes in liver tissues (Fig 6). The most significant effect of resveratrol was observed with *CYP1A2* and *CYP8B1* isoforms, since there was a considerable reduction in *CYP1A2* and induction in *CYP8B1* expression. At the same time, diabetic *CYP2A1*, *CYP2E1* and *CYP8B1* expression levels were almost normalized with resveratrol treatment.

### Immunoblot Analysis of SOD-1, SOD-2 and GST-Mu

**Fig 7:**
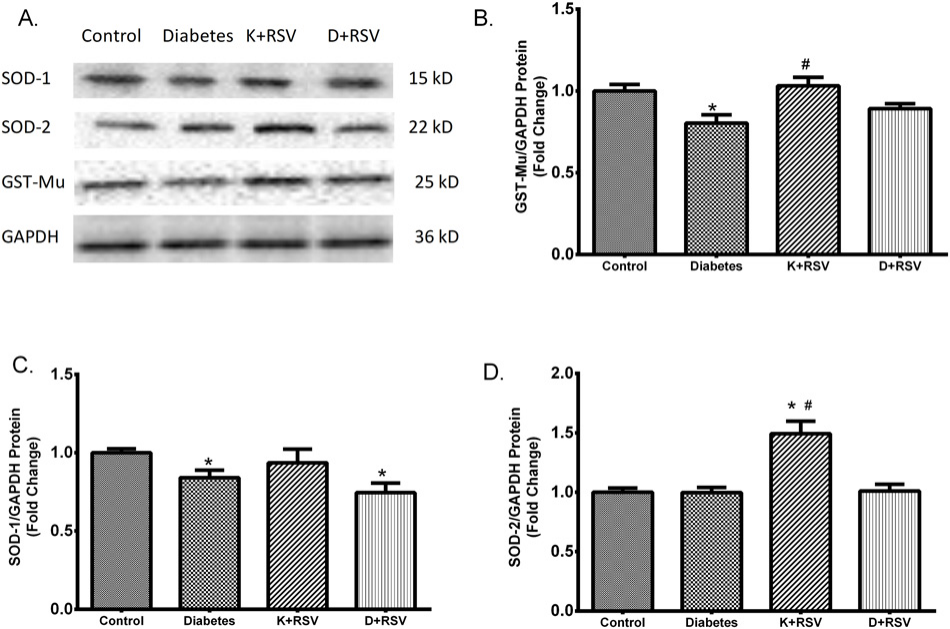
Protein contents of major antioxidant enzymes. Representative images for GST-Mu, SOD-1 and SOD-2 protein contents measured by Western blot analysis (A). The intensity of the bands was quantified using densitometry and normalized with corresponding GAPDH. Effects of diabetes and resveratrol on GST-Mu (B) SOD-1 (C) and SOD-2 (D) protein levels are summarized. Each bar represents at least six rats. *Indicates that the means were significantly different (p<0.05) compared to control groups, #Indicates that the means were significantly different (p<0.05) compared to diabetic groups. K+RSV: Resveratrol supplemented control group, D+RSV: Resveratrol supplemented diabetic group.

In this study, we also analyzed the protein levels of SOD-1, SOD-2 and GST-Mu and their concerted modulation in diabetic liver with or without resveratrol. Immunoblot results were summarized in representative blot images (Fig 7A). Densitometric analysis revealed that similar with the mRNA expression data, protein levels of SOD-1 and GST-Mu were also reduced in diabetic liver. GST-Mu and SOD-2 protein levels were affected from resveratrol in such a way that application to diabetic animals normalized GST-Mu towards control values (Fig 7B). This result was in agreement with mRNA expression of the same protein. Resveratrol induced SOD-2 levels in control group but any significant effect was observed on SOD-1 proteins (Fig 7C and 7D).

### Effect of Resveratrol on SOD-1, SOD-2, Total GST and GST-Mu Activities in Diabetic Liver

The transcription data is useful for identifying potential candidates for follow-up work at the protein and activity level. Gene expression data can suggest whether or not the protein is present or not and roughly what level to expect to see the protein level. Usually, a highly abundant protein has a highly expressed mRNA. However; in some cases, change in mRNA and protein levels do not correlate that well mainly due to the regulation at different levels. Therefore, biochemical functions of proteins and enzymes might not be correlated to their associated mRNA levels. On the basis of this, biochemical status of SOD-1, SOD-2, and GSTs (Total-GST and GST-Mu) were determined by measuring the enzymatic activities.

**Fig 8:**
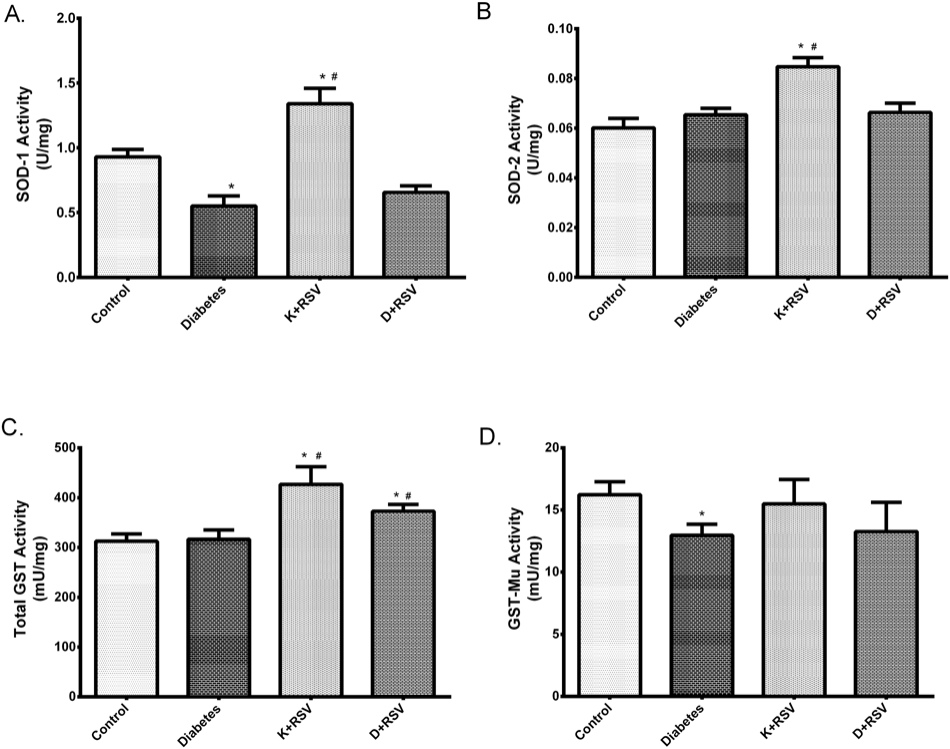
Summary of the changes in SOD-1 (A), SOD-2 (B), total GST (C) and GST-Mu (D) activities. Each bar represents at least nine rats. *Indicates that the means were significantly different (p<0.05) compared to control groups, #Indicates that the means were significantly different (p<0.05) compared to diabetic groups. K+RSV: Resveratrol supplemented control group, D+RSV: Resveratrol supplemented diabetic group.

It was observed that, diabetes decreased the activities of both SOD-1 and GST-Mu below the control values. SOD-1 and GST-Mu activities were decreased about 41% and 20%, respectively in diabetics (Fig 8). Repressions of these activities were in parallel with their mRNA and protein levels reflecting that transcriptional repression lead the enzymatic activities to be silenced. In addition, resveratrol increased SOD-1 and SOD-2; as well as total GST activity as given to control group. Conversely, except total GST, it did not affect the activities in STZ-induced diabetic group significantly.

## Discussion

Microarray is a high throughput technology giving a snapshot of all the transcriptional activity in a biological sample. Detection of gene expression profile by using microarray provides identification of underlying mechanisms of a disease or drug response. Diabetes is a metabolic disorder usually originated from the combination of genetic and environmental factors. It causes tissue damage by creating oxidative stress-induced pathologies in a number of tissues [31–33]. Deterioration of liver functions and the molecular basis of metabolic changes caused by diabetes have not been fully elucidated. The present study used a genome-wide expression approach to characterize alterations in liver gene expression by STZ-induced diabetes and exploit the role of resveratrol on gene expression patterns in rat liver tissues.

Resveratrol (3,4’,5-trihydroxystilbene) is a polyphenolic compound found mainly in grapes, wine, peanuts and blueberries. In addition to antioxidant [34], anti-inflammatory [35], anti-apoptotic properties [36], it has ability to protect against oxidative damage in the pathophysiology of diabetes [37,38]. At the same time, it affects expression levels of several apoptotic and anti-proliferative genes [39,40].

The results of this study provide another evidence for the presence of oxidative stress in the liver tissues of diabetic rats. It was confirmed with the increment in TOS and MDA levels and decrease in GSH/GSSH ratio by STZ administration. Normalization of TOS, MDA and GSH/GSSH ratio demonstrated strong antioxidant properties of resveratrol.

General appearance of microarray data revealed that gene expression regulatory/modulatory action of resveratrol was found to be stronger than STZ. Since, diabetes up-regulated 90 genes, which was the half number of genes, whose expression was augmented by resveratrol application. Similarly, while 183 genes were down-regulated with diabetes, resveratrol affected approximately 2.5-fold more (494) genes.

Diabetes-induced genes are responsible from catalytic activities, oxidation reduction reactions, co-enzyme and NADP binding and terpenoid biosynthesis processes. The largest change caused by diabetes occurred in *Usp2* gene. This gene is responsible from the production of enzymes having a role in the removal of ubiquitin molecules on variety of proteins to mark them to go through proteolysis. Furthermore, diabetes up-regulated the genes of phase I and II detoxification enzymes such as, *CYP2J4*, *CYP1A1*, *P450* (cytochrome) oxidoreductase, *Nat8*, *GCNT2*, *GSTYc2*. Such significant increases in these genes indicate the rise of diabetic detoxification processes which is also confirmed by pathway analysis.

Down-regulated genes with diabetes were mainly responsible from cellular metabolic processes. At the same time, genes in carbohydrate metabolism, regulation of transcription, intracellular signal transduction, calcium-dependent cell-to-cell adhesion and lipid catabolism were also suppressed. The most significant decrease occurred in *Npas2* gene chiefly involved in transcriptional control. In addition to *Npas2*, eight more transcription factors were also suppressed. In our recent study, we have demonstrated that expression levels of catalase and glutathione peroxidase were suppressed with diabetes [13]. *SOD-1*, *SOD-2*, *GST-Mu* gene expressions were also found to be reduced in this study. Therefore; decrease in the mRNA levels of aforementioned transcription factors in diabetes may be associated with decreased gene expression of antioxidant and some detoxification enzymes. Moreover, diabetes suppressed the genes responsible from carbohydrate metabolism which could be explained by the inability of blood glucose usage in the absence of insulin. A decrease in the expression of genes functioning in intracellular signal transduction is a sign of the impairment in cellular communication mechanisms.

When the effects of resveratrol on the gene expression profiles were analyzed in detail, the greatest impact is seen to occur in the genes providing the cellular integrity and continuity. In order to strengthen the cellular and cytoplasmic constituents, 150 genes were up-regulated by resveratrol. At the same time, resveratrol activated the genes forming responses to biotic stimulus and supported immune and defense mechanisms. The most noteworthy effect of resveratrol belonged to the expression of genes; *Lcn2* and *Usp2* functioning in the regulation of apoptosis. Resveratrol had also significant effects on *Akta1*, *Mylpf* and *Myl1* genes which are responsible from cellular motility and *S100a9* thought to be related to acute inflammation. All these changes caused by resveratrol elucidate its potential in reducing apoptotic responses and cell motility together with its immune-enhancing properties. Suppression of genes related to nucleus, nucleolus, nuclear envelope and nuclear core portions with resveratrol is well established. In addition to the *Lcn2* gene in the regulation of apoptosis (35.8 fold increase), reduction in the expression levels of *Pdcd4* (8.8 fold) and *Tp53bp2* (tumor protein p53 binding protein) genes (2.8-fold) acting as apoptosis inhibitors, refers to the ability of resveratrol in regulation of controlled cell death.

In addition to immune responses, resveratrol treatment to the diabetic animals also induced genes which are producing responses to internal and external stimuli and functioning in positive regulation of biological and developmental processes. The expression levels of genes exhibiting the greatest increase in D+RSV group were *Lcn2*, *Abcc3*, *GPx2*, *CYP1A1*, *CYP26A1*, *Igfbp2*, *GST-A5* and *Lgals3*. It is clear that an increase in the expression of these genes might be an indicator of the enhanced protection mechanisms for metabolic changes occurred as a result of diabetes, since most of these genes are functioning in detoxification metabolism.

A large portion of the down-regulated genes in resveratrol treated diabetic group are determined to be in charge from the synthesis of enzymes having catalytic activities in lipid homeostasis and sulfation reactions. Down-regulation of *Dhrs7* and *Car3* playing roles in response to oxidative stress; *CYP8B1* involving in the sterol metabolism, *Nox4* enabling the formation of reactive oxygen species have been well identified. Decreases in the expression level of these genes illustrate the contribution of resveratrol in the reduction of reactive oxygen species (ROS) and oxidative stress, and thus it may have positive effects against oxidative stress modifiers.

As given to the diabetic animals, resveratrol induced genes producing responses to abiotic, stress and chemical stimuli in liver tissues. Together with these increments, a rise in the expression level of genes encoding the components of the immune system might point out supportive functions of resveratrol in enhancing immune responses against inflammatory state. Resveratrol also down-regulated the genes of gluconeogenesis and glycogen catabolism in diabetic rat liver tissues. It is known that diabetic liver tissues deprive the glucose from the blood that might result in accelerated gluconeogenesis and glycogen breakdown. Down-regulation of those genes demonstrates the power of resveratrol in reducing the need for glucose in diabetic rat liver tissues.

According to pathway analysis, it is clear that STZ-induced diabetes mostly affected the xenobiotic metabolism. Additionally, pathways involved in chemical carcinogenesis, retinol metabolism and insulin signaling were also highly modulated. Resveratrol mostly modified the genes in the pathways related to cancer, chemokine, PI3K-AKT, MAPK signal transduction as well as cell cycle. In this context, resveratrol was found to suppress the genes involved in the carcinogenesis and appears to regulate the cell cycle. In addition, it led to significant changes in the PI3K-AKT pathway. While resveratrol is responsible from the activation of certain genes located in chemokine signal transduction, it also organizes the genes in cellular processes such as proliferation, differentiation, motility, responses to stress and apoptosis. The expression level of the genes, which are responsible from both activation and removal of xenobiotics from the body, significantly enhanced with resveratrol in diabetic group due to acceleration of detoxification process. Resveratrol also suppressed the expression levels of genes in steroid metabolism, NFκB signaling, retinol metabolism and some other signal transduction elements in diabetic animals. Reduction of these elements by resveratrol confirms its regulatory and/or normalizing effects on diabetes associated irregulatories.

The major antioxidant enzymes in cells are superoxide dismutase isozymes; SOD-1 and SOD-2 which protect radical induced tissue damage via reducing oxidative stress in cytoplasm and mitochondria, respectively. Another protective mechanism against oxidative stress is glutathione S-transferases (GSTs), which catalyze the conjugation of glutathione to a wide range of electrophiles. They exhibit differential response to a wide variety of chemicals and oxidative stress in the normal and pathophysiological conditions such as diabetes mellitus [25]. Among the different isoenzymes of GSTs, the cytosolic Mu and Pi isoenzymes function in the elimination of diverse array of foreign compounds as well as wide variety of products of oxidative stress [41]. In addition, cytochrome P450 enzymes are primarily responsible from the metabolism of endogenous and exogenous xenobiotics.

Recent studies indicated that oxidative stress might lead to the inactivation of abovementioned enzymes diminishing the organism's antioxidant defense system [42]. In relation with this depiction; we analyzed the expression levels of antioxidant (*SOD-1*, *SOD-2*, and *GST-Mu*) and detoxification enzymes (cytochrome P450s) in deep scanning with qRT-PCR. It was revealed that detoxification enzymes had trends towards up-regulation with diabetes while this situation for antioxidant enzymes was the reverse. Increase in the expression of particularly *CYP2E1* isozymes in diabetic animals, is seen as an adaptation for the elimination of oxidative products and the pathology of diabetes [43]. Down-regulation of the antioxidant and some detoxification enzymes might be explained by the decrease in transcription factors such as *NFκB* and *Nrf2* which might affect the transcription of those genes. Despite, diabetes reduced the expression levels of *Nrf2*, which is a redox-sensitive transcription factor, it induced *NFκB* [13]. Repression of *Nrf2* expression with diabetes and its normalization with resveratrol treatment demonstrated the down-regulation of *Nrf2* signaling with oxidative changes, which in turn affected downstream regulatory proteins, such as antioxidant and detoxification enzymes in the diabetic situation [10,13]. Microarray studies also revealed that diabetes decreased the expression levels of a number of other genes (9 genes) in the regulation of transcription. Suppression of these transcription factors could affect the levels of antioxidant and detoxification enzymes. Effects of resveratrol on antioxidant enzymes did not generally occur at the level of transcription but at the level of activity. Since, mRNA expression levels of antioxidant enzymes did not change in a significant way but increase in activities (SOD-1 and SOD-2) revealed the post-translational effect of resveratrol, which is the novel finding of the study.

To sum up, deep scanning of total mRNA expression of genes in experimental diabetic rat liver tissues were performed in this study. Besides, changes occurred by the strong antioxidant; resveratrol was also studied at transcriptome level. In addition to gene and protein expression analysis of antioxidant enzymes, their activity data enlighten the regulation mechanisms over these enzymes. Results demonstrated that resveratrol supplementation could be useful in the alleviation of streptozotocin-induced disturbances, which is evidenced by the normalization of the antioxidant and detoxification enzymes. Furthermore, transcriptome data obtained with the present study would facilitate a wide range of future studies to establish common and tissue-specific molecular mechanisms underlying the development of diabetic complications, with the aim of discovering new diagnostic markers or treatments with natural therapeutic compounds.

## Supporting Information

S1 Fig Regulated pathways. The top KEGG pathways significantly correlated with diabetes and resveratrol with associated genes and their fold changes. Colors in the figures demonstrate the fold change (FC) values as indicated in legends.
(RAR)

S2 Fig Western blot images. (PDF)

S1 File The ARRIVE guideline checklist for animal data. (PDF)

S1 Table Primer sequences used in qRT-PCR experiments. (PDF)

S2 Table Ontologically classified genes that were modulated with diabetes and resveratrol. Tables shows the list of ontologically categorized genes, whose expression levels were significantly up- and down-regulated with STZ or/and resveratrol treatment, with their probe numbers, fold change values as well as gene symbols.
(PDF)

## References

[1] Thirunavukkarasu M, Penumathsa SV, Koneru S, Juhasz B, Zhan L, Otani H, et al Resveratrol alleviates cardiac dysfunction in streptozotocin-induced diabetes: Role of nitric oxide, thioredoxin, and heme oxygenase. Free Radic Biol Med. 2007;43: 720–9. 10.1016/j.freeradbiomed.2007.05.004 17664136 PMC2586116

[2] Van Lunteren E, Moyer M, Spiegler S. Alterations in lung gene expression in streptozotocin-induced diabetic rats. BMC Endocr Disord. BMC Endocrine Disorders; 2014;14: 5 10.1186/1472-6823-14-5 24423257 PMC3945062

[3] Van Lunteren E, Moyer M. Gene expression of sternohyoid and diaphragm muscles in type 2 diabetic rats. BMC Endocr Disord. BMC Endocrine Disorders; 2013;13: 43 10.1186/1472-6823-13-43 24199937 PMC3851765

[4] Kume E, Aruga C, Ishizuka Y, Takahashi K, Miwa S, Itoh M, et al Gene expression profiling in streptozotocin treated mouse liver using DNA microarray. Exp Toxicol Pathol. 2005;56: 235–244. 10.1016/j.etp.2004.09.002 15816352

[5] Zhang F, Xu X, Zhang Y, Zhou B, He Z, Zhai Q. Gene expression profile analysis of type 2 diabetic mouse liver. PLoS One. 2013;8: e57766 10.1371/journal.pone.0057766 23469233 PMC3585940

[6] Pandey KB. Plant polyphenols as dietary antioxidants in human health and disease. Oxid Med Cell Longev. 2009;2: 270–278. 10.4161/oxim.2.5.9498 20716914 PMC2835915

[7] Szkudelski T, Szkudelska K. Anti-diabetic effects of resveratrol. Ann N Y Acad Sci. 2011;1215: 34–39. 10.1111/j.1749-6632.2010.05844.x 21261639

[8] Marques FZ, Markus MA, Morris BJ. Resveratrol: Cellular actions of a potent natural chemical that confers a diversity of health benefits. Int J Biochem Cell Biol. 2009;41: 2125–2128. 10.1016/j.biocel.2009.06.003 19527796

[9] Szkudelska K, Szkudelski T. Resveratrol, obesity and diabetes. Eur J Pharmacol. Elsevier B.V.; 2010;635: 1–8. 10.1016/j.ejphar.2010.02.054 20303945

[10] Palsamy P, Subramanian S. Resveratrol protects diabetic kidney by attenuating hyperglycemia-mediated oxidative stress and renal inflammatory cytokines via Nrf2-Keap1 signaling. Biochim Biophys Acta. Elsevier B.V.; 2011;1812: 719–31. 10.1016/j.bbadis.2011.03.008 21439372

[11] De la Lastra CA, Villegas I. Resveratrol as an antioxidant and pro-oxidant agent: mechanisms and clinical implications. Biochem Soc Trans. 2007;35: 1156–60. 10.1042/BST0351156 17956300

[12] Ji H, Wu L, Ma X, Ma X, Qin G. The effect of resveratrol on the expression of AdipoR1 in kidneys of diabetic nephropathy. Mol Biol Rep. 2014;41: 2151–9. 10.1007/s11033-014-3064-2 24413998

[13] Sadi G, Bozan D, Yildiz HB. Redox regulation of antioxidant enzymes: post-translational modulation of catalase and glutathione peroxidase activity by resveratrol in diabetic rat liver. Mol Cell Biochem. 2014;393: 111–22. 10.1007/s11010-014-2051-1 24740756

[14] Leon-Galicia I, Diaz-Chavez J, Garcia-Villa E, Uribe-Figueroa L, Hidalgo-Miranda A, Herrera LA, et al Resveratrol induces downregulation of DNA repair genes in MCF-7 human breast cancer cells. Eur J Cancer Prev. 2013;22: 11–20. 10.1097/CEJ.0b013e328353edcb 22644231

[15] Konings E, Timmers S, Boekschoten M V, Goossens GH, Jocken JW, Afman LA, et al The effects of 30 days resveratrol supplementation on adipose tissue morphology and gene expression patterns in obese men. Int J Obes (Lond). 2014;38: 470–3. 10.1038/ijo.2013.155 23958793

[16] Domínguez B, Pardo BG, Noia M, Millán A, Gómez-Tato A, Martínez P, et al Microarray analysis of the inflammatory and immune responses in head kidney turbot leucocytes treated with resveratrol. Int Immunopharmacol. 2013;15: 588–96. 10.1016/j.intimp.2013.01.024 23419489

[17] Jones SB, DePrimo SE, Whitfield ML, Brooks JD. Resveratrol-induced gene expression profiles in human prostate cancer cells. Cancer Epidemiol Biomarkers Prev. 2005;14: 596–604. 10.1158/1055-9965.EPI-04-0398 15767336 PMC3889115

[18] Kilkenny C, Browne WJ, Cuthill IC, Emerson M, Altman DG. Improving bioscience research reporting: the ARRIVE guidelines for reporting animal research. Osteoarthritis Cartilage. 2012;20: 256–60. 10.1016/j.joca.2012.02.010 22424462

[19] Zheng Q, Wang X-J. GOEAST: a web-based software toolkit for Gene Ontology enrichment analysis. Nucleic Acids Res. 2008;36: W358–63. 10.1093/nar/gkn276 18487275 PMC2447756

[20] Kanehisa M, Goto S. KEGG: kyoto encyclopedia of genes and genomes. Nucleic Acids Res. 2000;28: 27–30. Available: http://www.pubmedcentral.nih.gov/articlerender.fcgi?artid=102409&tool=pmcentrez&rendertype=abstract 10592173 10.1093/nar/28.1.27 PMC102409

[21] Ye J, Coulouris G, Zaretskaya I, Cutcutache I, Rozen S, Madden TL. Primer-BLAST: a tool to design target-specific primers for polymerase chain reaction. BMC Bioinformatics. 2012;13: 134 10.1186/1471-2105-13-134 22708584 PMC3412702

[22] Lowry O, Roserbrough N, Farr A, Randall R. Protein measurement with folin phenol reagent. J Biol Chem. 1951;193: 265–275. 14907713

[23] Towbin H, Staehelin T, Gordon J. Electrophoretic transfer of proteins from polyacrylamide gels to nitrocellulose sheets: procedure and some applications. Biotechnology. 1992;24: 145–9. Available: http://www.ncbi.nlm.nih.gov/pubmed/1422008 1422008

[24] Sadi G, Yilmaz O, Güray T. Effect of vitamin C and lipoic acid on streptozotocin-induced diabetes gene expression: mRNA and protein expressions of Cu-Zn SOD and catalase. Mol Cell Biochem. 2008;309: 109–16. 10.1007/s11010-007-9648-6 18008141

[25] Sadi G, Kartal DI, Guray T. Regulation of Glutathione S-Transferase Mu with type 1 diabetes and its regulation with antioxidants. Turkish J Biochem. 2013;38: 92–10. 10.5505/tjb.2013.96720

[26] Marklund S, Marklund G. Involvement of the superoxide anion radical in the autoxidation of pyrogallol and a convenient assay for superoxide dismutase. Eur J Biochem. 1974;47: 469–74. Available: http://www.ncbi.nlm.nih.gov/pubmed/4215654 4215654 10.1111/j.1432-1033.1974.tb03714.x

[27] Habig WH, Pabst MJ, Jakoby WB. Glutathione S-transferases. The first enzymatic step in mercapturic acid formation. J Biol Chem. 1974;249: 7130–9. Available: http://www.ncbi.nlm.nih.gov/pubmed/4436300 4436300

[28] Baur JA. Resveratrol, sirtuins, and the promise of a DR mimetic. Mech Ageing Dev. 2010;131: 261–9. 10.1016/j.mad.2010.02.007 20219519 PMC2862768

[29] Zhang J. Resveratrol inhibits insulin responses in a SirT1-independent pathway. Biochem J. 2006;397: 519–27. 10.1042/BJ20050977 16626303 PMC1533305

[30] Kolesnikov N, Hastings E, Keays M, Melnichuk O, Tang YA, Williams E, et al ArrayExpress update-simplifying data submissions. Nucleic Acids Res. 2014;43: D1113–6. 10.1093/nar/gku1057 25361974 PMC4383899

[31] Schmatz R, Perreira LB, Stefanello N, Mazzanti C, Spanevello R, Gutierres J, et al Effects of resveratrol on biomarkers of oxidative stress and on the activity of delta aminolevulinic acid dehydratase in liver and kidney of streptozotocin-induced diabetic rats. Biochimie. Elsevier Masson SAS; 2012;94: 374–83. 10.1016/j.biochi.2011.08.005 21864646

[32] Sadi G, Eryilmaz N, Tütüncüoğlu E, Cingir Ş, Güray T. Changes in expression profiles of antioxidant enzymes in diabetic rat kidneys. Diabetes Metab Res Rev. 2012;28: 228–35. 10.1002/dmrr.1302 22057777

[33] Bree AJ, Puente EC, Daphna-Iken D, Fisher SJ. Diabetes increases brain damage caused by severe hypoglycemia. Am J Physiol Endocrinol Metab. 2009;297: E194–201. 10.1152/ajpendo.91041.2008 19435850 PMC2711670

[34] Vlachogianni IC, Fragopoulou E, Kostakis IK, Antonopoulou S. In vitro assessment of antioxidant activity of tyrosol, resveratrol and their acetylated derivatives. Food Chem. 2015;177: 165–73. 10.1016/j.foodchem.2014.12.092 25660873

[35] Ghanim H, Sia CL, Abuaysheh S, Korzeniewski K, Patnaik P, Marumganti A, et al An antiinflammatory and reactive oxygen species suppressive effects of an extract of Polygonum cuspidatum containing resveratrol. J Clin Endocrinol Metab. 2010;95: E1–8. 10.1210/jc.2010-0482 20534755 PMC2936054

[36] Signorelli P, Ghidoni R. Resveratrol as an anticancer nutrient: molecular basis, open questions and promises. J Nutr Biochem. 2005;16: 449–66. 10.1016/j.jnutbio.2005.01.017 16043028

[37] Brasnyó P, Molnár G a, Mohás M, Markó L, Laczy B, Cseh J, et al Resveratrol improves insulin sensitivity, reduces oxidative stress and activates the Akt pathway in type 2 diabetic patients. Br J Nutr. 2011;106: 383–9. 10.1017/S0007114511000316 21385509

[38] Venturini CD, Merlo S, Souto AA, Fernandes MDC, Gomez R, Rhoden CR. Resveratrol and red wine function as antioxidants in the nervous system without cellular proliferative effects during experimental diabetes. Oxid Med Cell Longev. 2010;3: 434–441. 10.4161/oxim.3.6.14741 21307644 PMC3154048

[39] Harati K, Slodnik P, Chromik AM, Goertz O, Hirsch T, Kapalschinski N, et al Resveratrol induces apoptosis and alters gene expression in human fibrosarcoma cells. Anticancer Res. 2015;35: 767–74. Available: http://www.ncbi.nlm.nih.gov/pubmed/25667456 25667456

[40] Chin Y-T, Hsieh M-T, Yang S-H, Tsai P-W, Wang S-H, Wang C-C, et al Anti-proliferative and gene expression actions of resveratrol in breast cancer cells in vitro. Oncotarget. 2014;5: 12891–907. Available: http://www.pubmedcentral.nih.gov/articlerender.fcgi?artid=4350334&tool=pmcentrez&rendertype=abstract 25436977 10.18632/oncotarget.2632 PMC4350334

[41] Raza H. Dual localization of glutathione S-transferase in the cytosol and mitochondria: implications in oxidative stress, toxicity and disease. FEBS J. 2011;278: 4243–51. 10.1111/j.1742-4658.2011.08358.x 21929724 PMC3204177

[42] Seven A, Guzel S, Seymen O, Civelek S, Bolayirli M, Uncu M, et al Effects of vitamin E supplementation on oxidative stress in streptozotocin induced diabetic rats: investigation of liver and plasma. Yonsei Med J. 2004;45: 703–10. Available: http://www.ncbi.nlm.nih.gov/pubmed/15344213 15344213 10.3349/ymj.2004.45.4.703

[43] Arinç E, Arslan S, Bozcaarmutlu A, Adali O. Effects of diabetes on rabbit kidney and lung CYP2E1 and CYP2B4 expression and drug metabolism and potentiation of carcinogenic activity of N-nitrosodimethylamine in kidney and lung. Food Chem Toxicol. 2007;45: 107–18. 10.1016/j.fct.2006.07.026 17034923

