# Supplementary material for: Differential Gene Expression in Liver Tissues of Streptozotocin-Induced Diabetic Rats in Response to Resveratrol Treatment
Source: PLoS One. 2015 Apr 23;10(4):e0124968. doi: 10.1371/journal.pone.0124968 (PMC4408020; doi:10.1371/journal.pone.0124968)
Supplement: S2 Fig — (PDF) [file pone.0124968.s002.pdf]

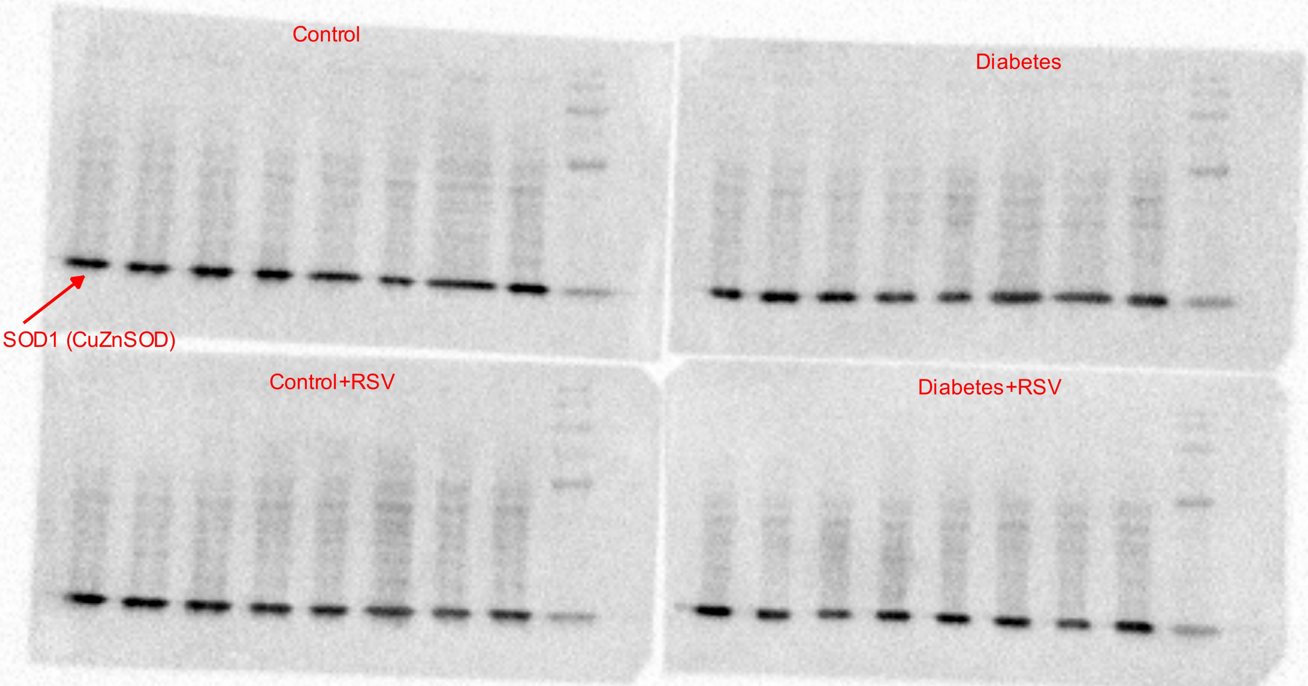
Control
Diabetes
SOD1 (CuZnSOD)
Control+RSV
Diabetes+RSV

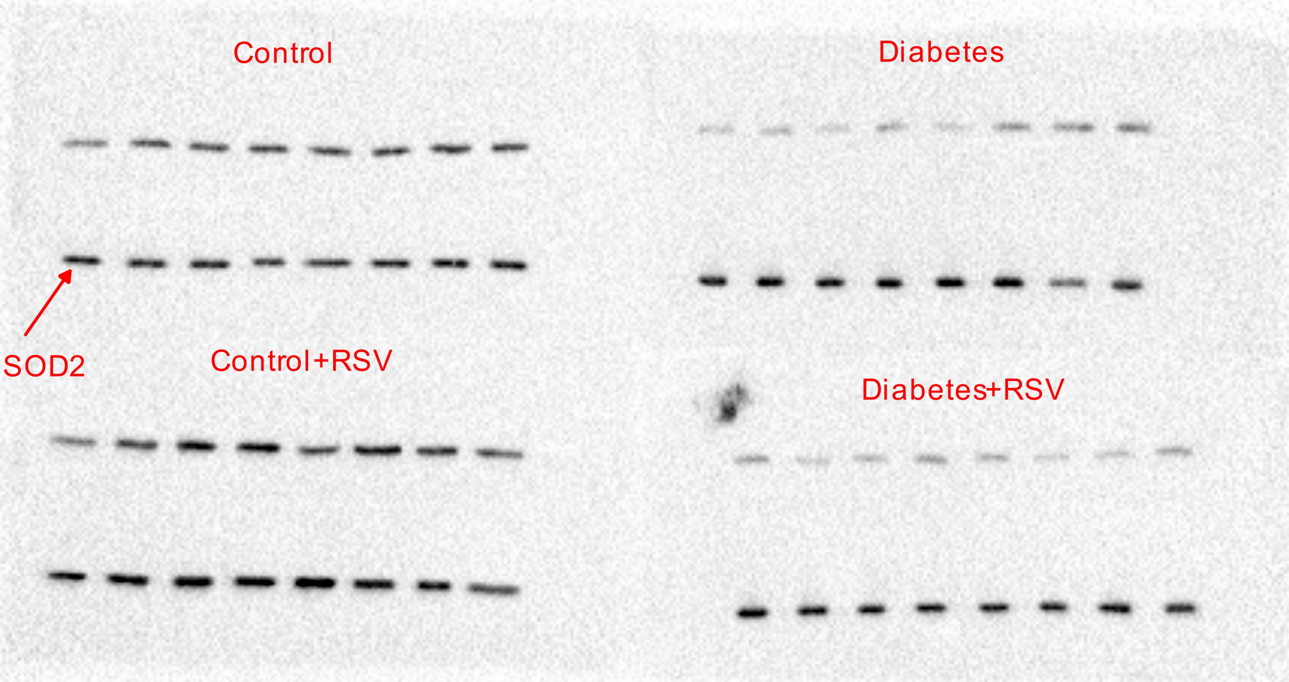
Control
Diabetes
SOD2
Control+RSV
Diabetes+RSV

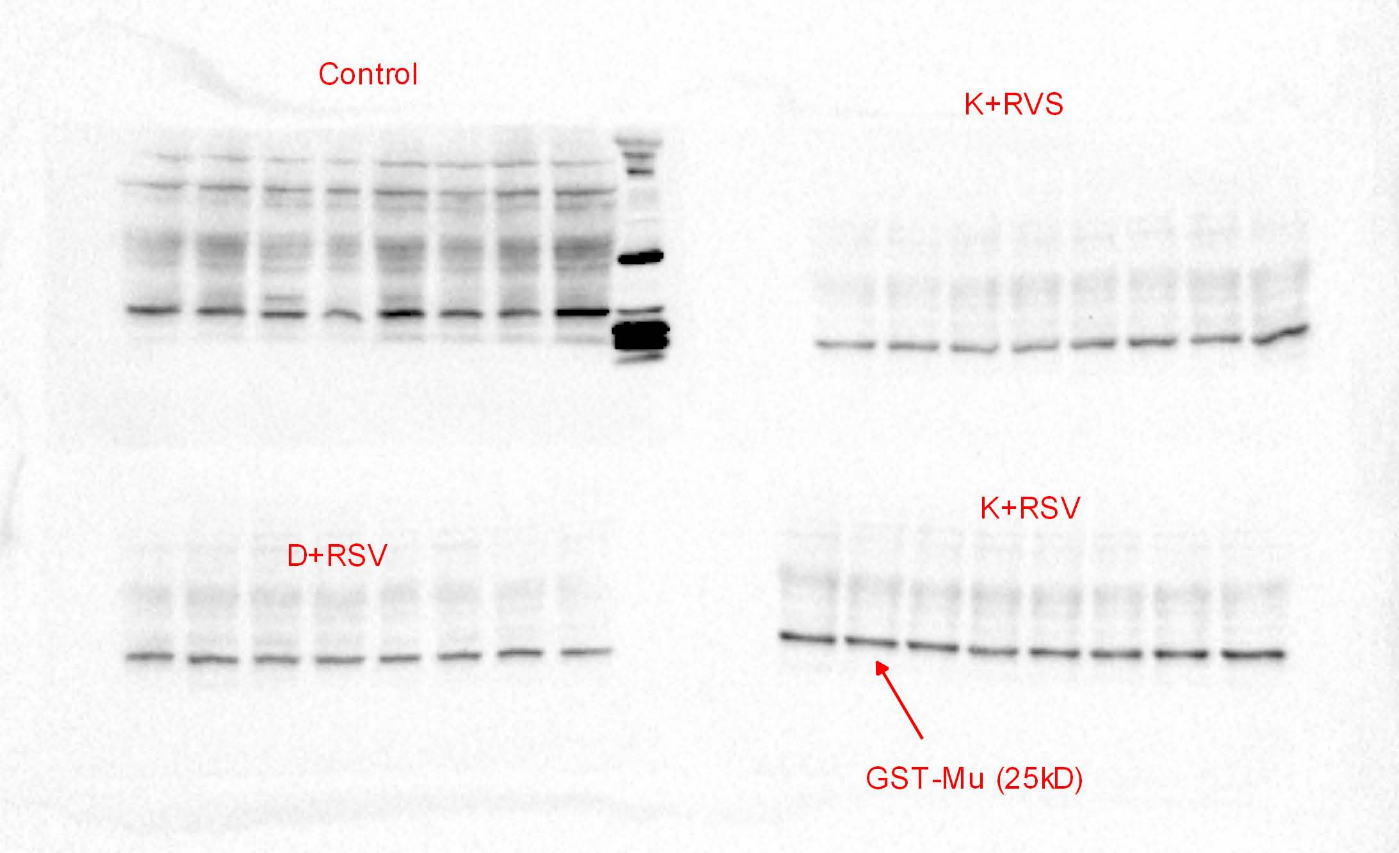
Control
K+RVS
D+RSV
K+RSV
GST-Mu (25kD)

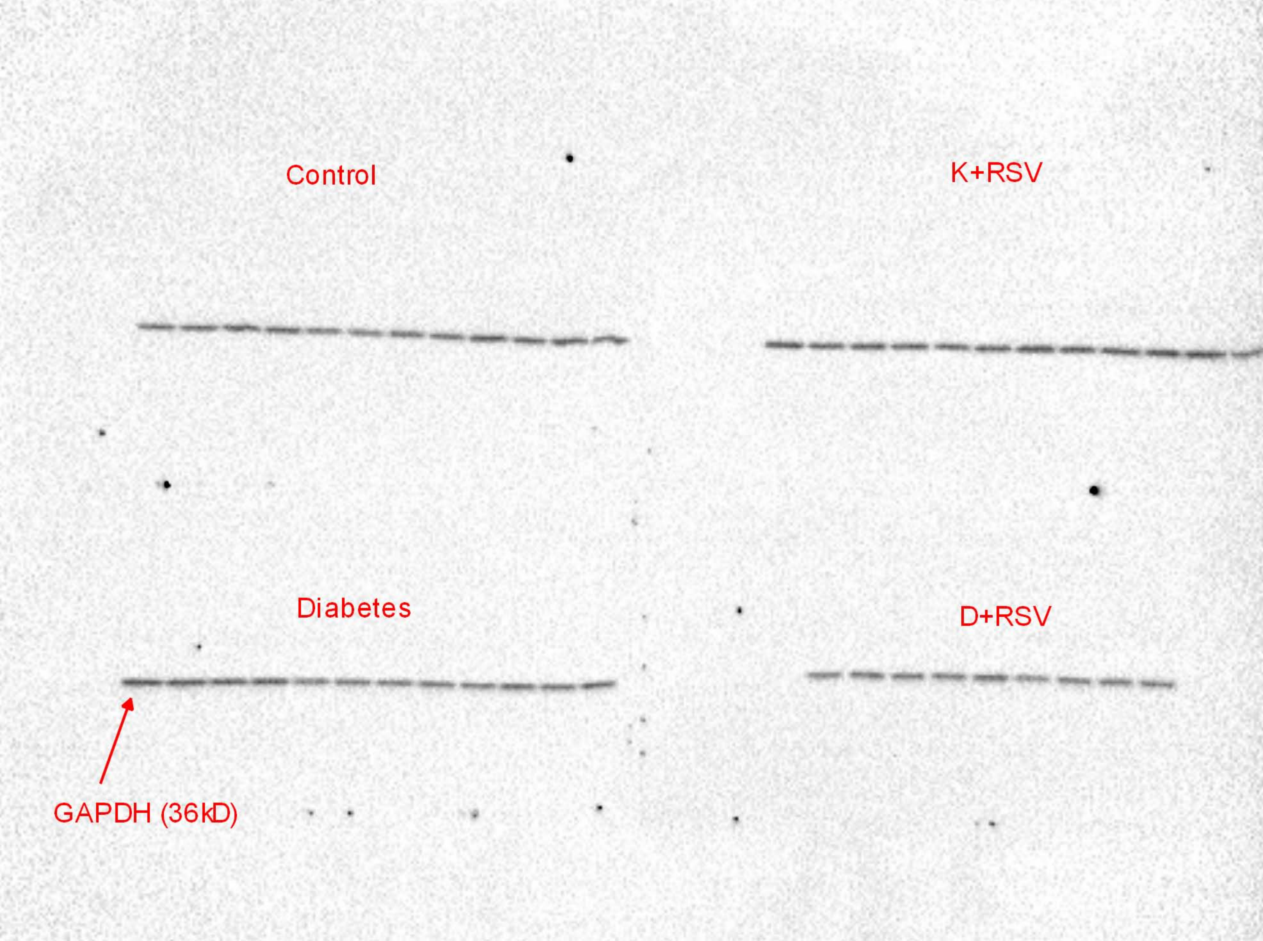
Control
K+RSV
Diabetes
D+RSV
GAPDH (36kD)

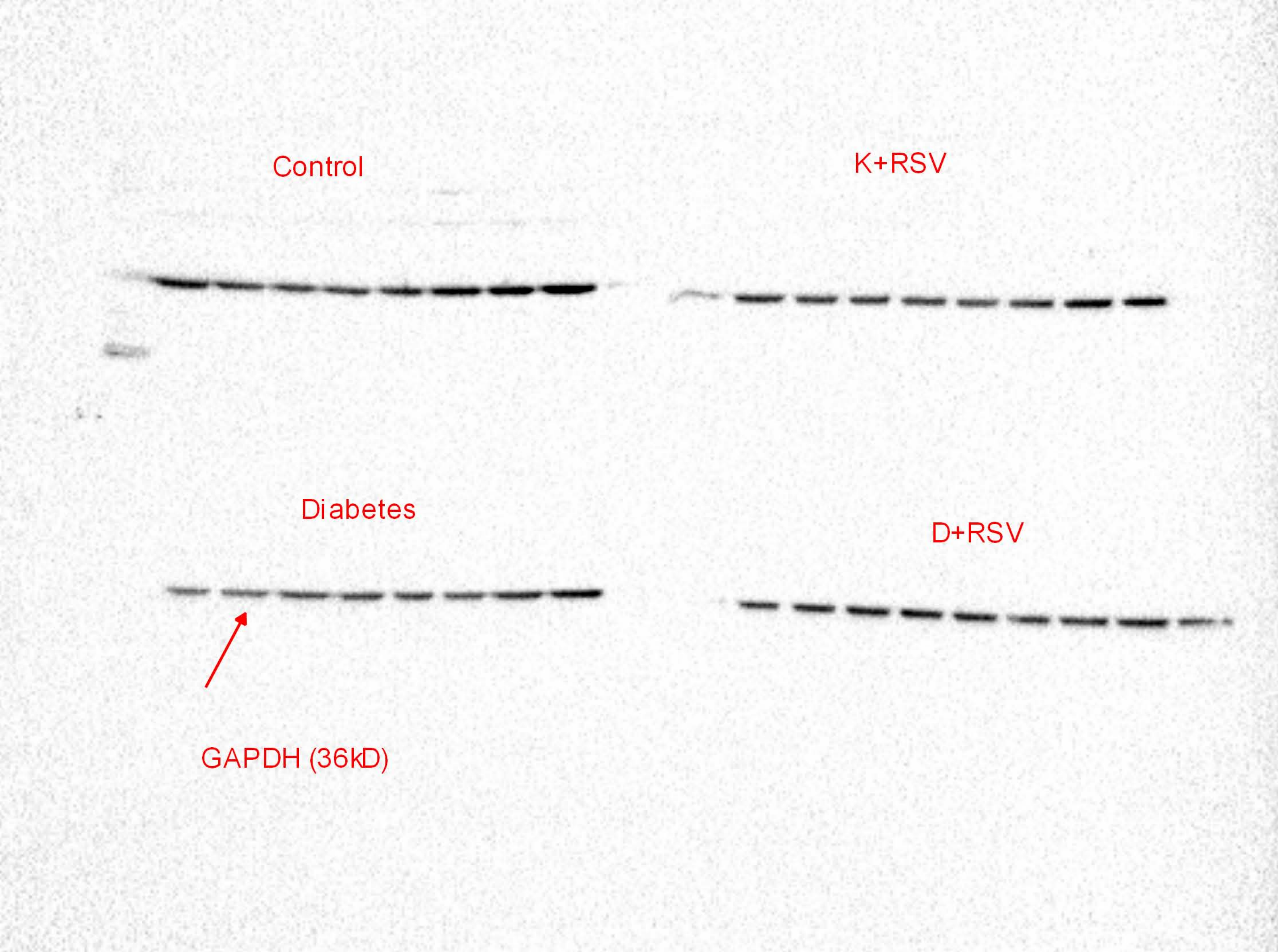
Control
K+RSV
Diabetes
D+RSV
GAPDH (36kD)
