# Supplementary material for: Differential Gene Expression in Liver Tissues of Streptozotocin-Induced Diabetic Rats in Response to Resveratrol Treatment
Source: PLoS One. 2015 Apr 23;10(4):e0124968. doi: 10.1371/journal.pone.0124968 (PMC4408020; doi:10.1371/journal.pone.0124968)
Supplement: S1 Table — (PDF) [file pone.0124968.s004.pdf]

Table 1. Primer sequences used for qRT-PCR.

Gene	Forward Primer (5'→3')	Reverse Primer (5'→3')
GAPDH	TCCTTGGAGGCCATGTGGGCCAT	TGATGACATCAAGAAGGTGGTGAAG
SOD-1	TAGCAGGACAGCAGATGAGT	GCAGAAGGCAAGCGGTGAAC
SOD-2	GCACATTAACGCGCAGATCA	AGCCTCCAGCAACTCTCCTT
GST-Pi	TTCGTCCACTACTGTTTACC	CCTCACCCTTTACCAATCTA
GST-Mu	AGAAGCAGAAGCCAGAGTTC	GGGGTGAGGTTGAGGAGATG
CYP1A1	CTGGTTCTGGATACCCAGCTG	CCTAGGGTTGGTTACCAGG
CYP1A2	GTCACCTCAGGGAATGCTGTG	GTTGACAATCTTCTCCTGAGG
CYP2A1&2	CACAGGGCAGCTCTATGACA	CAGACCCAGCAAAGAAGAGG
CYP2B1&2	GAGTTCTTCTCTGGGTTCCTG	ACTGTGGGTCATGGAGAGCTG
CYP2C11	CTGCTGCTGCTGAAACACGTG	GGATGACAGCGATACTATCAC
CYP2E1	CTCCTCGTCATATCCATCTG	GCAGCCAATCAGAAATGTGG
CYP4A1	GGTGACAAAGAACTACAGC	AGAGGAGTCTTGACCTGCCAG
CYP8B1	GCCCCCAGTGAGATGAAGAC	TAAGGCAGGTAGGATGGGCT
Cyp8b1	TAAGGCAGGTAGGATGGGCT	GCCCCCAGTGAGATGAAGAC
Usp2	GCACAGAGCTCCTTACGTGT	CTCAACGGAGGCAGTGGATT
GstA5	CACGATTCTCGCTAGTGAAGC	CCATGGGCACTTGGTCAAAC
Igfbp2	GCCATGCTTGTCACAGTTGG	TTACGCTGTTACCCCAACCC
