# Supplementary material for: Differential Gene Expression in Liver Tissues of Streptozotocin-Induced Diabetic Rats in Response to Resveratrol Treatment
Source: PLoS One. 2015 Apr 23;10(4):e0124968. doi: 10.1371/journal.pone.0124968 (PMC4408020; doi:10.1371/journal.pone.0124968)
Supplement: S2 Table — Tables shows the list of ontologically categorized genes, whose expression levels were significantly up- and down-regulated with STZ or/and resveratrol treatment, with their probe numbers, fold change values as well as gene symbols. (PDF) [file pone.0124968.s005.pdf]

Table 1. Ontologically classified genes whose expression levels were at least 2-fold up-regulated with diabetes and their fold change values.

Probe Set ID	Gene Symbol	Entrez gene number	Gene Ontology	Fold Change (FC)
**Catalytic Activity**				
1387703_a_at	Usp2	115771	Deubiquitinating enzyme, removes ubiquitin from covalent attachments to itself or other proteins	15,97
1387336_at	Nat8	64570	May act as an N-acetyltransferase	7,87
1369467_a_at	Pfkfb1	24638	Fructose-2,6-biphosphatase bifunctional enzyme; catalyzes synthesis and degradation of fructose 2,6-bisphosphate	5,08
1371089_at	Gsta5	494500	Glutathione S-transferase Yc2 subunit	4,69
1383665_at	Lpin2	316737	Lipin 2	4,06
1370663_at	Wee1	308937	Wee 1 homolog	3,09
1392994_at	LOC499330	499330	Nicotinamide riboside kinase 1	2,98
1374903_at	Gcnt2	306860	Glucosaminyl (N-acetyl) transferase 2	2,86
1389014_at	Nampt	297508	Has similarity to cytokines may be associated with the cell cycle	2,85
1397409_s_at	Wee1	308937	Wee 1 homolog	2,82
1396300_at	Afmid	688283	Arylformamidase	2,73
1370436_at	Acsm2	246263	May bind AMP; may be involved in kidney function	2,65
1370991_at	Cml3	113892	May act as an N-acetyltransferase	2,65
1396301_x_at	Afmid	688283	Arylformamidase	2,56
1392640_at	Cry1	299691	A negative regulator of the CLOCK:BMAL1-driven feedback loop which controls circadian rhythms	2,52
1368127_at	Neu2	29204	Cytosolic enzyme that cleaves sialic acid; involved in the degradation of glycolipids and glycoproteins	2,49
1372038_at	Mknk2	299618	MAP kinase-interacting serine/threonine kinase 2	2,39
1387659_at	Gda	83585	Exhibits guanine deaminase activity	2,29
1387809_at	Map2k6	114495	Activator of p38 MAPK; may play a role in cardiac hypertrophic and failure conditions	2,23
1394479_at	Afmid	688283	Arylformamidase	2,19
1372533_at	Edem1	297504	ER degradation enhancer, mannosidase alpha-like 1	2,06
1370592_at	Keg1	171179	Hepatocellular carcinoma-enriched gene	2,03
1370938_at	Pdxp	727679	Pyridoxal (pyridoxine, vitamin B6) phosphatase	2,02
**Terpenoid Biosnthesis**				
1370269_at	Cyp1a1	24296	Monooxygenase that plays a role in dioxin metabolism	2,99
1378260_at	Adh1	24172	Class I alcohol dehydrogenase; metabolizes a wide variety of substrates including ethanol, hydroxysteroids and lipid peroxidation products	2,85

1387022_at	Aldh1a1	24188	Ubiquitous enzyme catalyzes oxidation of aldehyde substrates to carboxylic acids; detoxifies ethanol-derived acetaldehyde	2,81
1375852_at	Hmgcr	25675	Enzyme involved in mevalonate synthesis	2,36
1387296_at	Cyp2j4	65210	Cytochrome p450 monooxygenase enzyme; may be involved with rat model of pneumonia	2,24

NADP Binding

1387109_at	Por	29441	P450 (cytochrome) oxidoreductase	4,70
1368213_at	Por	29441	Plays a role in electron transport	2,34
1377635_at	Fmo2	246245	Encodes a catalytically inactive	2,12

Coenzyme Binding

1374555_at	Acbd6	289125	Acyl-coa binding domain containing 6	2,30
1368283_at	Ehhadh	171142	Enzyme involved in the B-oxidation cycle of peroxisomes	2,09

Oxidation-Reduction Reactions

1368121_at	Akr7a3	26760	Aldo-keto reductase family 7	2,09
1398252_at	Mecr	29470	İnteracts with PPAR and with various nuclear hormone receptors	2,07

Table 2. Ontologically classified genes whose expression levels were at least 2-fold down-regulated with diabetes and their fold change values.

Probe Set ID	Gene Symbol	Entrez gene number	Gene Ontology	Fold Change (FC)
**Ca^{++} Independent Cell-to-Cell Binding**				
1375933_at	Cldn2	300920	Claudin 2	-2,41
1387470_at	Cldn1	65129	Claudin 1	-2,06
1396150_at	Cldn1	65129	Claudin 1	-2,06
**Cellular Carbohydrates Catalytic Processes**				
1370725_a_at	G6pc	25634	Glucose-6-phosphatase, catalyzes the conversion of D-glucose 6-phosphate to D-glucose	-3,74
1386944_a_at	G6pc	25634	Glucose-6-phosphatase, catalyzes the conversion of D-glucose 6-phosphate to D-glucose	-2,68
1368674_at	Pygl	64035	Phosphorylase, glycogen catalyzes the rate-limiting step of glycogen catabolism	-2,16
1368674_at	Pygl	64035	Carbohydrate metabolic process	-2,16
1371251_at	Galt	298003	Galactose metabolic process	-2,73
1373108_at	Ppp1r3c	309513	Regulation of glycogen biosynthetic process	-3,09
1386944_a_at	G6pc	25634	Glucose 6-phosphate metabolic process	-2,68
1386977_at	Car3	54232	One-carbon metabolic process, response to oxidative stress	-2,30
1387139_at	Hao2	84029	Mandelate metabolic process	-2,29
1391661_at	Gk	79223	Glycerol-3-phosphate metabolic process	-2,74
**Lipid Metabolisms**				
1368426_at	Crot	83842	Medium-chain fatty acid transport	-2,69
1389377_at	Insig2	288985	Response to fatty acid	-2,60
**Regulation of Transcription**				
1368870_at	Id2	25587	Negative regulation of transcription from RNA polymerase II promoter	-2,32
1370816_at	Nr1d1	252917	Regulation of transcription, DNA-dependent	-4,12
1370928_at	Litaf	65161	Positive regulation of I-kappab kinase/NF-kappab cascade	-2,29
1372601_at	Atf5	282840	Regulation of transcription, DNA-dependent	-2,06
1381967_at	Rbm39	362251	Mrna processing	-2,32
1398759_at	Tsc22d1	498545	Regulation of transcription, DNA-dependent	-2,23
1380027_at	Crebbp	54244	Regulation of transcription, DNA-dependent	-2,31
1383439_at	Npas2	316351	Regulation of transcription, DNA-dependent	-16,00
1390907_at	LOC691170	691170	Regulation of transcription, DNA-dependent	-2,04

Cellular Signal Transduction

1368779_a_at	Gucy1b2	25206	cGMP biosynthetic process	-2,30
1369044_a_at	Pde4b	24626	cAMP catabolic process	-3,99
1374204_at	Wsb1	303336	İntracellular signaling pathway	-3,35
1375796_at	Igtp	303163	GTP catabolic process	-2,26
1376788_at	Dapk1	306722	Protein phosphorylation	-2,07
1387981_at	Olr59	170816	G-protein coupled receptor protein signaling pathway	-2,73
1382020_at	Spag9	360600	Activation of MAPK activity	-2,23
1395403_at	Stac3	362895	İntracellular signaling pathway	-7,19
1397304_at	Igtp	303163	GTP catabolic process	-2,97

Table 3. Ontologically classified genes whose expression levels were at least 2-fold up-regulated with resveratrol and their fold change values.

Probe Set ID	Gene Symbol	Entrez gene number	Gene Ontology	Fold Change (FC)
**Immune Response**				
1387011_at	Lcn2	170496	Plays a role in IL3 withdrawal-induced apoptosis	35,80
1387787_at	Mylpf	24584	Myosin light chain	5,94
1369964_at	Coro1a	155151	Actin binding protein and may be involved in mitosis	2,87
1370219_at	Cyba	79129	Cytochrome b558, a component of NAD(P)H oxidase	2,69
1388602_at	Cfd	54249	Serine protease adipocyte-specific protein	2,49
1367712_at	Timp1	116510	TIMP metallopeptidase inhibitor 1	2,46
1373025_at	C1qc	362634	C1qa complement component 1	2,40
1369031_at	Il18bp	84388	Antagonist for the proinflammatory responses induced by endogenous IL-18	2,34
1370904_at	RT1-DMa	294274	MHC molecule involved in antigen presentation and can serve as a molecular chaparone	2,32
1370119_at	Lst1	64569	İmmune-related gene located in the Class III region of the MHC	2,32
1370882_at	RT1-DMb	294273	May play a role in antigen presentation	2,31
1378015_at	Ccl21b	298006	Chemokine (C-C motif) ligand 21	2,30
1379760_at	Tnfaip8l2	310663	Tumor necrosis factor	2,28
1387835_at	Il1rn	60582	Binds to IL-1 receptor, blocking the binding of IL-1 alpha and -beta	2,27
1369029_at	Plscr1	117540	May act as a downstream mediator of immune response in ıge receptor signaling	2,27
1377943_at	Cd86	56822	Plays a role in T-cell activation and proliferation	2,23
1367974_at	Anxa3	25291	İnhibits phospholipase A2 activity	2,14
1390687_at	Plek	364206	Pleckstrin	2,14
1370443_at	Dnase2a	171575	Catalyzes the hydrolysis of DNA into oligonucleotides under acidic conditions	2,11
1392280_at	Tlr2	310553	Toll-like receptor 2	2,10
1370215_at	C1qb	29687	Complement protein involved in innate immune response	2,08
1398246_s_at	Fcgr2a /// LOC498276	116591///498276	Receptor that binds immunoglobulin gamma	2,04
1376652_at	C1qa	298566	C1qa complement component 1	2,04
**Defense Response**				
1387125_at	S100a9	94195	Calcium binding protein that may be associated with acute inflammatory processes	4,89
1367847_at	Nupr1	113900	Nuclear proten 1	2,75

1378032_at	Nfkbiz	304005	Nuclear factor of kappa light polypeptide gene enhancer in B-cells inhibitor	2,73
1369204_at	Hck	25734	Hemopoietic cell kinase	2,72
1368693_at	Fgr	79113	Phosphorylates phospholipase D2 (PLD2); plays a role in mast cell activation	2,65
1389873_at	Pycard	282817	May play a role in apoptosis	2,60
1388596_at	Cotl1	361422	Coactosin-like 1 (Dictyostelium)	2,20
1369672_at	Alox5ap	29624	İnvolved in leukotriene synthesis; plays a role in immediate hypersensitivity response	2,06
**Response to Stress**				
1368877_at	Zfp354a	24522	C2H2 class zinc finger-containing DNA-binding protein	4,09
1390507_at	Isg20	293052	İnterferon stimulated exonuclease gene 20	3,95
1369735_at	Gas6	58935	Provides protection of neurons against serum deprivation-induced apoptosis	2,73
1387925_at	Asns	25612	Crucial for asparagine synthesis; may be important for progression through the G1 phase of the cell cycle	2,54
1374070_at	Gpx2	29326	Glutathione peroxidase 2 which functions in the detoxification of hydrogen peroxide	2,47
1367734_at	Akr1b1	24192	Regulates NF-kappa B mediated mitogenic signaling; may play a role in the polyol pathway	2,28
1369693_a_at	Slc1a2	29482	Solute carrier family 1 (glial high affinity glutamate transporter)	2,28
1374303_at	Alkbh2	304578	Alkb, alkylation repair homolog 2	2,19
1369467_a_at	Pfkfb1	24638	Fructose-2,6-biphosphatase; catalyzes synthesis and degradation of fructose 2,6-bisphosphate	2,19
1368007_at	Dmbt1	170568	Deleted in malignant brain tumors 1	2,12
1392784_at	Gas6	58935	Provides protection of neurons against serum deprivation-induced apoptosis	2,12
1367577_at	Hspb1	24471	Heat shock protein 1	2,07
1369931_at	Pkm2	25630	Pyruvate kinase; catalyzes the conversion of ATP and pyruvate to ADP and	2,07
1370023_at	Gja4	25655	Gap junction protein that mediates cell-cell communication	2,02
1372013_at	Ifitm1	293618	İnterferon induced transmembrane protein 1	2,00
1369590_a_at	Ddit3	29467	Plays a role in the ER stress response	2,00
**Response to Organic Compounds**				
1369928_at	Acta1	29437	Actin, may play a role in muscle function	24,76
1388433_at	Krt19	360626	Keratin 19	2,62
1382026_at	Arhgap9	362893	Rho gtpase activating protein 9	2,52
1379295_at	Gngt2	690825	Guanine nucleotide binding protein	2,23
1379812_at	Nnmt	300691	Nicotinamide N-methyltransferase	2,12
1374284_at	Rassf4	362423	Ras association (ralgds/AF-6) domain family member 4	2,08
1374649_at	Rasgrp2	361714	RAS guanyl releasing protein 2	2,05
1367660_at	Fabp3	79131	Binds fatty acids; may play a role in fatty acid metabolism	2,01

Positive Regulation of Biological Process

1372016_at	Gadd45b	299626	Growth arrest and DNA-damage-inducible	4,12
1374033_at	Psmb10	291983	Weakly similar to proteasome subunit, beta type	3,43
1383665_at	Lpin2	316737	Lipin 2	2,91
1368376_at	Nr0b2	117274	İnteracts with peroxisome proliferator-activated receptor alpha	2,65
1387109_at	Por	29441	P450 (cytochrome) oxidoreductase	2,29
1383013_at	Klf13	499171	Moderately similar to BTE1 RAT TRANSCRIPTION FACTOR	2,28
1372404_at	Rac2	366957	Ras-related C3 botulinum toxin substrate 2 (rho family, small GTP binding protein Rac2)	2,16
1367816_at	Hopx	171160	HOP homeobox	2,14
1391791_at	Acer2	313339	Alkaline ceramidase 2	2,11
1368370_at	Adcy4	54223	Plays a role in Gs alpha and G-protein beta gamma mediated signaling	2,08
1387343_at	Cebpd	25695	Ccaatenhancerbinding, protein (CEBP) delta	2,06
1370371_a_at	Ceacam1 /// Ceacam10	287009///81613	Cell-cell adhesion molecule	2,04
1389606_at	Anapc1	311412	Anaphase promoting complex subunit 1	2,04

Cytoplasm

1387703_a_at	Usp2	115771	Deubiquitinating enzyme, removes ubiquitin from covalent attachments to itself or other proteins	7,41
1370033_at	Myl1	56781	Myosin, light chain 1	4,04
1372691_at	Upp1	289801	Uridine phosphorylase 1	3,60
1370436_at	Acsm2	246263	May bind AMP; may be involved in kidney function	3,52
1377034_at	Serpinb1a	291091	Serine (or cysteine) proteinase inhibitor	2,83
1378252_at	Chodl	288289	Chondrolectin	2,67
1389409_at	Tes	500040	Testis derived transcript	2,62
1370503_s_at	Epb4.1l3	116724	Human homolog acts as a tumor suppressor and binds 14-3-3 proteins	2,60
1370516_at	Slc15a3	246239	May play a role in protein catabolism in the lymphatic system	2,48
1368505_at	Rgs4	29480	Regulates G-protein coupled receptor	2,48
1368006_at	Laptm5	89783	A lysosomal protein involved in microglial activation	2,46
1377016_at	Creld2	362978	Cysteine-rich with EGF-like domains 2	2,42
1388112_at	Slc25a4	85333	Catalyzes the exchange of ADP and ATP across the mitochondrial inner membrane	2,32
1379766_at	Sla	338477	An adaptor protein; may be involved in the signaling of receptor tyrosine kinases	2,28
1383401_at	Tes	500040	Testis derived transcript	2,17
1370147_at	Acmsd	171385	Aminocarboxymuconate semialdehyde decarboxylase	2,16
1392701_at	Gmppb	363145	GDP-mannose pyrophosphorylase B	2,16
1367925_at	Mvp	64681	Member of a large cytosolic ribonucleoprotein particle; may play a role in vault function	2,14

1367846_at	S100a4	24615	May play a role in regulation of cell growth and differentiation	2,13
1376431_at	Sil1	291673	Human homolog acts as a nucleotide exchanger; may play a role in protein folding in the endoplasmic reticulum	2,13
1387658_at	Eef2k	25435	Catalyzes the phosphorylation of eukaryotic elongation factor 2	2,12
1371817_at	Isyna1	290651	İnositol-3-phosphate synthase 1	2,09
1368164_at	Blvra	116599	Catalyzes the conversion of biliverdin to bilirubin in heme degradation	2,09
1384759_at	Phf17	310352	PHD finger protein 17	2,07
1388403_at	Idh2	361596	İsocitrate dehydrogenase 2 (NADP+), mitochondria	2,07
1370817_at	Sec11c	266758	Subunit of the signal peptidase complex, which cleaves the signal sequences of secretory and membrane proteins	2,06
1380621_at	Fes	361597	Feline sarcoma oncogene	2,06
1367722_at	Dpp7	83799	Catalyses the hydrolysis of N-terminal dipeptides Xaa-Xbb-|-Xcc	2,05
1370020_at	Slc25a10	170943	Transport protein found in the inner mitochondrial membranes	2,03
1375654_at	Ckap4	362859	Cytoskeleton-associated protein 4	2,02

Cell Components

1370150_a_at	Thrsp	25357	Expression is induced by dietary sucrose and by thyroid hormone	3,28
1371400_at	Thrsp	25357	Expression is induced by dietary sucrose and by thyroid hormone	3,22
1371247_at	Tnnt3	24838	Plays a role in muscle contraction	2,63
1387808_at	Slc7a7	83509	Subunit of the y+LAT-1 amino acid transporter	2,32
1374851_at	Med29	292751	Mediator complex subunit 29	2,23
1372468_at	Cd97	361383	CD97 molecule	2,18
1387795_at	Pola2	85242	Plays a role in DNA replication	2,15
1374249_at	RGD1304580	292781	Spectrin repeat containing,	2,14
1370108_a_at	Lin7a	85327	May mediate the formation of cell-cell junctions	2,11
1373590_at	Stom	296655	Stomatin	2,10
1373668_at	Polr2i	292778	Polymerase (RNA) II	2,07
1372219_at	Tpm2	500450	Tropomyosin 2, beta	2,04
1372135_at	LOC684352	684352	Similar to twinfilin-like protein	2,01

Extracellular region

1388460_at	Capg	297339	Capping protein (actin filament), gelsolin-like	2,66
1387794_at	Fcn1	83517	Plasma protein that binds elastin and glcnac	2,57
1393467_at	Pcsk5	116548	May play a role in peptide processing and maturation	2,31
1389911_at	Metrnl	316842	Meteorin, glial cell differentiation regulator-like	2,14
1386879_at	Lgals3	83781	Lectin molecule that binds ıge; may have role in immune function	2,04
1373607_at	St3gal3	64445	Plays a role in the formation of terminal carbohydrate groups of glycolipids and glycoproteins	2,04

Table 4. Ontologically classified genes whose expression levels were at least 2-fold down-regulated with resveratrol and their fold change values.

Probe Set ID	Gene Symbol	Entrez gene number	Gene Ontology	Fold Change (FC)
**Nucleoulus**				
1393459_at	Fmr1	24948	May play a role in mrna trafficking and localization	-6,09
1374299_at	Dhx9	304859	DEAH (Asp-Glu-Ala-His) box helicase 9	-5,24
1386950_at	Ppp1cb	25594	İsoforms of protein phosphatase 1	-4,91
1391786_at	Hnrnph2	308650	Heterogeneous nuclear ribonucleoprotein H2	-4,33
1381925_x_at	Arid1b	282546	AT rich interactive domain 1B transcription factor; involved in the response to acute and chronic renal ischemic stress	-4,18
1383410_at	Srp54a	116650	May recognize signal sequences of secretory proteins	-3,79
1383336_at	Pnn	368070	Pinin, desmosome associated protein	-3,26
1398595_at	Rbm5	300996	RNA binding motif protein 5	-3,21
1383720_at	Utx	100310845	Lysine (K)-specific demethylase 6A	-2,95
1394107_at	Tbl1xr1	365755	Transducin (beta)-like 1 X-linked receptor 1	-2,78
1375901_at	Ddx21	317399	DEAD (Asp-Glu-Ala-Asp) box helicase 21	-2,71
1388799_at	Klhl7	362303	Kelch-like family member 7	-2,67
1367619_at	Pgrmc1	291948	Selectively binds odorants and may be involved in carrying odorants	-2,67
1384101_at	Wasl	682507	İnvolved in the regulation of the cortical actin cytoskeleton	-2,47
1375378_at	Qk	499022	Quaking	-2,45
1374752_at	Mdfic	362325	Myod family inhibitor domain containing	-2,40
1383377_at	Gabpa	363735	GA binding protein transcription factor	-2,34
1394591_at	Zfp207	303763	Zinc finger protein 207	-2,27
1368212_at	Sfrs11	502603	Serine/arginine-rich splicing factor 11	-2,24
1368588_at	Ddx52	85432	DEAD (Asp-Glu-Ala-Asp) box polypeptide 52may act as an RNA helicase	-2,24
1368308_at	Myc	24577	Myelocytomatosis oncogene that plays a role in cell cycle progression, apoptosis and cellular transformation.	-2,19
1380283_at	Tshz1	307217	Teashirt zinc finger homeobox 1	-2,14
1373916_at	Ep300	170915	E1A binding protein p300 endogenous transcriptional coactivator related to fatty acid and vitamin A metabolism	-2,14
1377224_at	Taf1	317256	TAF1 RNA polymerase II, TATA box binding protein (TBP)-associated factor	-2,12
1392180_at	Sp1	24790	Transcription factor that recognizes 5'-CCGCCC promoter sequence	-2,12
1398883_at	Hnrnpa2b1	362361	Heterogeneous nuclear ribonucleoprotein A2/B1	-2,10

1377609_at	Hipk1	365895	Homeodomain interacting protein kinase 1	-2,05
1381998_at	Zfp148	58820	Zinc finger protein 148 regulates the transcription of genes such as gastrin, beta enolase	-2,00

Nuclear Lumen with Envelope

1392912_at	Cacybp	289144	Calcyclin binding protein	-7,71
1380447_a_at	Mrps18c	289469	Mitochondrial ribosomal protein S18C	-5,64
1376811_a_at	Cpsf6	299811	Cleavage and polyadenylation specific factor 6	-4,48
1389528_s_at	Jun	24516	Jun proto-oncogene transcription factor; acts as a protooncogene	-3,72
1368426_at	Crot	83842	Catalyzes the conversion of L-carnitine and coa; plays a role in fatty acid transport	-3,67
1392979_at	Cacybp	289144	Calcyclin binding protein	-3,38
1369737_at	Crem	25620	Camp responsive element modulator	-3,36
1385408_at	Mga	499874	MGA, MAX dimerization protein	-3,28
1370934_at	Nup153	25281	Nucleoporin 153 nuclear pore complex protein; may act to to gate transcribable genes to nuclear pore complexes	-3,14
1392502_at	Ahctf1	360886	AT hook containing transcription factor 1	-3,12
1370908_at	Hdac2	84577	Histone deacetylase 2 may be involved in chromatin rearrangement during neural differentiation	-3,12
1383439_at	Npas2	316351	Neuronal PAS domain protein 2	-2,90
1367627_at	Gatm	81660	Glycine amidinotransferase transamidinase enzyme that catalyzes creatine synthesis	-2,88
1388210_at	Acot2	192272	Acyl-coa thioesterase 2 hydrolase may have a role in lipid metabolism	-2,79
1393033_at	Yars2	287924	Tyrosyl-trna synthetase 2	-2,71
1393041_at	Smc2	362519	Structural maintenance of chromosomes 2	-2,64
1398940_at	Srrm2	302969	Serine/arginine repetitive matrix 2	-2,58
1374170_at	Ttrap	498749	Tyrosyl-DNA phosphodiesterase 2	-2,47
1379982_at	Nrip1	304157	Nuclear receptor interacting protein 1	-2,45
1368325_at	Egf	25313	Epidermal growth factor may play a role in MAP kinase mediated signaling pathways	-2,44
1390373_at	Smad5	59328	SMAD family member 5 may play a role in the healing of bone fractures	-2,29
1383098_at	Fyttd1	360726	Forty-two-three domain containing 1	-2,21
1379567_at	Med13	303403	Mediator complex subunit 13	-2,18
1371695_at	Tpr	304862	Translocated promoter region, nuclear basket protein	-2,15
1373916_at	Ep300	170915	E1A binding protein p300 endogenous transcriptional coactivator related to fatty acid and vitamin A metabolism	-2,14
1368992_a_at	Sfrs5	29667	Serine/arginine-rich splicing factor 5 may play a role in cell cycle regulation	-2,14
1372046_at	Gtf3c3	316810	General transcription factor IIIC, polypeptide 3	-2,11
1367995_at	Cat	24248	Catalase functions as a hydrogen peroxide:hydrogen peroxide reductase	-2,10

1379346_at	Cdc73	304832	Cell division cycle 73	-2,05
1392990_at	Sox17	312936	SRY (sex determining region Y)-box 17	-2,01
1393798_at	Atrx	246284	Binds to annexin V and may play a role in annexin V mediated activities	-2,01
1389827_at	Fdx1	29189	Ferredoxin 1	-2,01

Nucleus Division

1374318_at	Brcc3	316794	BRCA1/BRCA2-containing complex, subunit 3	-2,73
1385592_at	Bcor	317346	BCL6 co-repressor	-2,71
1383629_a_at	Rnpc3	691538	RNA-binding region (RNP1, RRM) containing 3	-2,62
1383172_at	Ranbp2	294429	RAN binding protein 2	-2,51

Nucleus

1383326_a_at	Pdcd4	64031	Programmed cell death 4 acts as an inhibitor of apoptosis	-8,79
1368924_at	Ghr	25235	May play a role in chondrocyte differentiation and positive regulation of longitudinal bond growth	-6,54
1393351_at	Rdh10	353252	Retinol dehydrogenase involved in the photic visual	-6,25
1391387_s_at	Slbp	681062	Stem-loop binding protein	-6,01
1379850_at	Psmc6	289990	Proteasome (prosome, macropain) 26S subunit	-5,83
1384323_at	Psmc6	289990	Proteasome (prosome, macropain) 26S subunit	-5,70
1387029_at	Cfh	155012	Complement factor H	-5,33
1393684_at	Hells	294071	Helicase, lymphoid specific	-5,01
1390820_at	Zcchc11	313481	Zinc finger, CCHC domain containing 11	-4,85
1383046_at	Cfh	155012	Complement factor H	-4,79
1374695_at	Cbx1	360609	Chromobox homolog 1	-4,04
1367515_at	Cnot7	306492	CCR4-NOT transcription complex, subunit 7	-3,84
1383965_at	Pigz	689116	Phosphatidylinositol glycan anchor biosynthesis	-3,67
1375880_at	Appbp2	303396	Amyloid beta precursor protein binding protein 2	-3,48
1370381_at	Pnrc1	286988	Proline-rich nuclear receptor coactivator 1	-3,46
1377820_a_at	Ate1	293526	Arginyltransferase 1	-3,37
1376587_at	Fbxo11	301674	F-box protein 11	-3,37
1387242_at	Eif2ak2	54287	Eukaryotic translation initiation factor 2-alpha kinase 2	-3,14
1389312_at	Memo1	298787	Mediator of cell motility 1	-3,14
1385639_at	Casp8ap2	313128	Caspase 8 associated protein 2	-3,06
1393701_at	Dbr1	681234	Debranching enzyme homolog 1	-3,01
1388737_at	Pbrm1	306254	Polybromo 1	-2,99
1398573_at	Zkscan3	306977	Zinc finger with KRAB and SCAN domains 3	-2,96
1383981_at	Tp53bp2	305025	Tumor protein p53 binding protein	-2,77
1375870_a_at	Rbms1	362138	RNA binding motif, single stranded interacting protein 1	-2,74
1391560_at	Hivep1	117140	Human immunodeficiency virus type I enhancer binding	-2,72

			protein 1	
1394086_at	Senp7	288167	SUMO1/sentrin specific peptidase 7	-2,68
1374548_at	Aff4	303132	AF4/FMR2 family, member 4	-2,61
1382741_at	Ube3a	361585	Ubiquitin protein ligase E3A	-2,60
1392599_at	Syap1	302678	Synapse associated protein 1	-2,42
1377907_at	Snrnp48	291060	Small nuclear ribonucleoprotein	-2,40
1391475_at	Hnrpll	313842	Heterogeneous nuclear ribonucleoprotein L-like	-2,35
1393267_at	Psip1	313323	PC4 and SFRS1 interacting protein 1	-2,33
1384352_at	Papd4	361878	PAP associated domain containing 4	-2,30
1373812_at	Cdkn1b	83571	Cyclin-dependent kinase inhibitor 1B	-2,28
1379723_at	RGD1565584	293112	CREB/ATF bzıp transcription factor	-2,27
1372785_at	Ash1l	310638	Ash1 (absent, small, or homeotic)-like	-2,20
1398888_at	H3f3b	117056	H3 histone, family 3B	-2,15
1372201_at	Ggnbp2	360584	Gametogenetin binding protein 2 may play a role in spermatogenesis	-2,12
1394022_at	Id4	291023	İnhibitor of DNA binding 4	-2,11
1399101_at	Rbm39	362251	RNA binding motif protein 39	-2,08
1397203_at	Prpf4b	291078	PRP4 pre-mrna processing factor 4 homolog B	-2,08
1395601_at	Nudt12	367323	Nudix (nucleoside diphosphate linked moiety X)-type motif 12	-2,08
1384217_at	Zhx2	314988	Zinc fingers and homeoboxes 2	-2,07
1368627_at	Rgn	25106	Regucalcin binds calcium ions	-2,06
1374602_at	Tspyl1	29544	TSPY-like 1	-2,06
1372360_at	Abi1	79249	Abl-interactor 1	-2,05
1390278_at	Tra2a	500116	Transformer 2 alpha homolog	-2,05
1382114_at	Tlk1	311118	Tousled-like kinase 1	-2,03
1389333_at	Fbxo3	690634	F-box protein 3	-2,02
1379027_at	Wwc1	303039	WW and C2 domain containing 1	-2,02
1385194_at	Rb1cc1	312927	RB1-inducible coiled-coil 1	-2,00

Table 5. Ontologically classified genes up-regulated by resveratrol in diabetic group (i.e. D+RSV vs Diabetes) and their fold change values.

Probe Set ID	Gene Symbol	Entrez gene number	Gene Ontology	Fold Change (FC)
**Immune Response**				
1387011_at	Lcn2	170496	Mouse homolog plays a role in IL3 withdrawal-induced apoptosis	23,26
1367712_at	Timp1	116510	TIMP metallopeptidase inhibitor 1	4,24
1367850_at	Fcgr2a	498276	Fc gamma receptor II beta	3,46
1398246_s_at	Fcgr2a /// LOC498276	116591///498276	Receptor that binds immunoglobulin gamma	3,30
1382106_at	Ccl6	287910	Chemokine (C-C motif) ligand 6	3,16
1383131_at	Itgb2	309684	İntegrin, beta 2	3,04
1389123_at	Ccl6	287910	Chemokine (C-C motif) ligand 6	2,96
1370215_at	C1qb	29687	Complement protein involved in innate immune response	2,89
1367974_at	Anxa3	25291	İnhibits phospholipase A2 activity	2,86
1398482_at	Bcl3	680611	B-cell CLL/lymphoma 3	2,81
1369029_at	Plscr1	117540	May act as a downstream mediator of immune response in ıge receptor signaling	2,79
1373025_at	C1qc	362634	C1qa complement component 1	2,76
1393347_at	Itgal	308995	The integrin alpha L chain which combines with beta 2 chain to form LFA-1	2,74
1373575_at	Fcer1g	25441	Subunit of high-affinity receptor for immunoglobulin E	2,73
1370219_at	Cyba	79129	Cytochrome b558, a component of NAD(P)H oxidase	2,63
1368482_at	Bcl2a1d	170929	BCL2-related protein A1	2,53
1376652_at	C1qa	298566	C1qa complement component 1	2,38
1398256_at	Il1b	24494	İnterleukin 1 beta	2,33
1368731_at	Orm1	24614	Orosomucoid 1 an acute phase reactant protein; may have a role in the acute inflammation response	2,27
1370119_at	Lst1	64569	İmmune-related gene located in the Class III region of the MHC	2,21
1370882_at	RT1-DMb	294273	May play a role in antigen presentation	2,21
1370904_at	RT1-DMa	294274	MHC molecule involved in antigen presentation and can serve as a molecular chaparone	2,19
1377943_at	Cd86	56822	Plays a role in T-cell activation and proliferation	2,11
1393411_at	Cfp	299314	Complement factor properdin	2,04
**Response to Other Organisms**				
1369698_at	Abcc3	140668	ATP-binding cassette, may play a role in steroid metabolism	11,20
1374070_at	Gpx2	29326	Glutathione peroxidase 2 which functions in the detoxification of hydrogen peroxide	6,60

1370269_at	Cyp1a1	24296	Cytochrome P450 monooxygenase that plays a role in dioxin metabolism	6,57
1387125_at	S100a9	94195	Calcium binding protein that may be associated with acute inflammatory processes	3,33
1369204_at	Hck	25734	Hemopoietic cell kinase	3,02
1388596_at	Cotl1	361422	Coactosin-like 1 (Dictyostelium)	2,92
1368374_a_at	Ggt1	116568	Gamma-glutamyltransferase 1	2,75
1375951_at	Thbd	83580	Thrombomodulin cell surface glycoprotein; plays an important role in the protein C anticoagulant pathway	2,15
1387995_a_at	Ifitm3	361673	İnterferon induced transmembrane protein 3	2,12
1370249_at	Tspo	24230	Benzodiazapene receptor that may be involved in the nenoatal reponse to hypoxia	2,11
1387247_at	Pcsk1	25204	Proprotein convertase subtilisin/kexin type 1 plays a role in proteolytic processing of peptide hormone and other protein precursors	2,09

Response to Stimuli

1387583_at	Cyp26a1	154985	Cytochrome P450, acts on retinoids, including all-trans-retinoic acid	6,58
1384243_at	Spsb4	300950	Spla/ryanodine receptor domain and SOCS box containing 4	3,58
1388784_at	Csf1r	307403	Colony stimulating factor 1 receptor may mediate normal and neoplastic growth of muscular cells	2,89
1370371_a_at	Ceacam1 /// Ceacam10	287009///81613	Cell-cell adhesion molecule	2,83
1384392_at	Cyp26b1	312495	Cytochrome P450 that catalyzes the inactivation of all-trans-retinoic acid to hydroxylated forms	2,74
1374649_at	Rasgrp2	361714	RAS guanyl releasing protein 2	2,59
1368025_at	Ddit4	140942	DNA-damage-inducible transcript 4 may protect H2O2-triggered apoptosis	2,54
1368905_at	Ces2l	171118	Carboxylesterase 2C enzyme may play a role in lipid metabolism	2,50
1378032_at	Nfkbiz	304005	Nuclear factor of kappa light polypeptide gene enhancer in B-cells inhibitor	2,37
1374730_at	Tyrobp	361537	Tyro protein tyrosine kinase binding protein	2,22
1381203_at	Sh3glb1	292156	SH3-domain GRB2-like endophilin B1	2,18
1382026_at	Arhgap9	362893	Rho gtpase activating protein 9	2,17
1367633_at	Glul	24957	Glutamate-ammonia ligase	2,14
1386870_at	Glul	24957	Glutamate-ammonia ligase	2,10
1387794_at	Fcn1	83517	Plasma protein that binds elastin and glcnac	2,03

Response to Lipids

1367648_at	Igfbp2	25662	İnsulin-like growth factor binding protein 2	5,58
1371143_at	Serpina7	81806	Serpin peptidase inhibitor acts as a carrier for thyroid hormone	5,15
1368376_at	Nr0b2	117274	İnteracts with peroxisome proliferator-activated receptor alpha (PPAR alpha)	4,75
1371298_at	H19	309122	H19, imprinted maternally expressed transcript	3,78

1368007_at	Dmbt1	170568	Malignant brain tumors 1	3,29
1388433_at	Krt19	360626	Keratin 19	2,73
1368259_at	Ptgs1	24693	Prostaglandin-endoperoxide synthase may be involved in methamphetamine neurotoxicity	2,59
1391791_at	Acer2	313339	Alkaline ceramidase 2	2,24
1387260_at	Klf4	114505	Kruppel-like factor 4 a transcription factor that works with Sp1	2,08
1370583_s_at	Abcb1a /// Abcb1b	170913///24646	ATP-binding cassette ATP-binding cassette	2,01

Response to External Stimuli

1371089_at	Gsta5	494500	Glutathione S-transferase	3,57
1387925_at	Asns	25612	Crucial for asparagine synthesis	2,87
1387675_at	Plau	25619	May play a role in tumor invasion and metastasis	2,83
1372404_at	Rac2	366957	Ras-related C3 botulinum toxin substrate 2 (rho family, small GTP binding protein Rac2)	2,12

Membrane Components

1386879_at	Lgals3	83781	Lectin molecule that binds ıge; may have role in immune function	3,92
1368778_at	Slc6a6	29464	Solute carrier family 6 acts as a sodium and chloride dependent taurine transporter	3,73
1387687_at	Igsf6	171064	İmmunoglobulin superfamily, member 6 human homologue may help in dimerizing through the transmembrane region	3,33
1376390_at	Ms4a11	361735	Membrane-spanning 4-domains, subfamily A	3,29
1368052_at	Tspan8	171048	Tetraspanin 8 metastasis-associated tetraspanin molecule; may play a role in thrombotic disorders	3,14
1387799_at	Fxyd2	29639	FXYD domain-containing ion transport regulator 2	3,12
1375010_at	Cd68	287435	Cd68 molecule	2,95
1390510_at	Ms4a6b	293749	Membrane-spanning 4-domains, subfamily A	2,68
1388460_at	Capg	297339	Capping protein (actin filament)	2,58
1378184_at	Cd200r2	501779	CD200 receptor 1-like	2,58
1368518_at	Cd53	24251	Cd53 molecule a tetraspanin protein; may be involved in cell survival and growth regulation	2,39
1370516_at	Slc15a3	246239	May play a role in protein catabolism in the lymphatic system	2,39
1367661_at	S100a6	85247	S100 calcium binding protein A6 calcium-binding protein involved in transepithelial ion transport	2,34
1387808_at	Slc7a7	83509	The y+LAT-1 amino acid transporter	2,32
1387005_at	Ctss	50654	Cathepsin S lysosomal cysteine proteases; may mediate matrix degradation, may play a role in thyroid hormone biosynthesis	2,29
1398373_at	B3galnt1	310508	Beta-1,3-N-acetylgalactosaminyltransferase 1	2,19
1368006_at	Laptm5	89783	A lysosomal protein involved in microglial activation	2,19
1370202_at	Pla2g16	24913	Phospholipase A2 gene whose expression is suppressed by HRAS	2,14
1388628_at	Tmed3	300888	Transmembrane emp24 protein transport domain containing	2,13

			3	
1390161_at	Cyyr1	304138	Cysteine/tyrosine-rich 1	2,13
1390672_at	Rprm	680110	Reprimo, TP53 dependent G2 arrest mediator candidat	2,00

Positive Regulation of Biological Processes

1372016_at	Gadd45b	299626	Growth arrest and DNA-damage-inducible	4,50
1387343_at	Cebpd	25695	Ccaatenhancerbinding, protein (CEBP) delta	2,81
1389179_at	Cidea	291541	Cell death-inducing DFFA-like effector a	2,32
1388199_at	Epcam	171577	Epithelial cell adhesion molecule involved in metastasis formation and tumor progression	2,02

Endocytosis

1385635_at	Cd5l	310693	Cd5 molecule-like	3,38
1386843_at	Cd5l	310693	Cd5 molecule-like	2,91
1368755_at	Clec4f	114598	C-type lectin domain family 4 carbohydrate binding receptor with an affinity for fucose and galactose	2,81
1393917_at	Cd163	312701	Scavenger receptor for the hemoglobin-haptoglobin complex	2,77

Single Cell Growth Processes

1367655_at	Tmsb10	50665	Thymosin actin-sequestering protein; binds actin monomers and inhibits actin polymerization	2,54
1367816_at	Hopx	171160	HOP homeobox	2,54
1368073_at	Irf1	24508	İnterferon regulatory factor 1 stimulates cell proliferation and regulates growth-inhibitory interferon genes	2,22

Table 6. Ontologically classified genes down-regulated by resveratrol in diabetic group (i.e. D+RSV vs Diabetes) and their fold change values.

Probe Set ID	Gene Symbol	Entrez gene number	Gene Ontology	Fold Change (FC)
**Single Cell Metabolic Processes**				
1397205_at	Dhrs7	299131	Dehydrogenase/reductase (SDR family) member 7	-17,73
1386977_at	Car3	54232	Carbonic anhydrase 3 catalyzes hydration of carbon dioxide; may be involved in cellular response to oxidative stress	-9,04
1367896_at	Car3	54232	Carbonic anhydrase 3 catalyzes hydration of carbon dioxide	-7,42
1368435_at	Cyp8b1	81924	Cytochrome P450 member; plays a role in sterol metabolism	-6,66
1368520_at	Apoa4	25080	Apolipoprotein A-IV may play a role in lipid transport	-6,41
1387156_at	Hsd17b2	79243	Hydroxysteroid (17-beta) dehydrogenase 2 17-beta hydroxysteroid enzyme that regulates the biological activity of sex hormones	-5,80
1368543_at	Nox4	85431	NADPH oxidase 4 enzyme involved in the production of reactive oxygen species in vascular smooth muscle cells	-4,99
1369864_a_at	Sds	25044	Serine dehydratase enzyme that converts L-serine to pyruvate and ammonia; involved in gluconeogenesis	-4,08
1368426_at	Crot	83842	Catalyzes the conversion of L-carnitine and octanoyl-coa to L-octanoylcarnitine and coa; plays a role in fatty acid transport	-3,76
1387139_at	Hao2	84029	Hydroxyacid oxidase 2 FMN-dependent alpha-hydroxy-oxidase enzyme that catalyzes the oxidation of L-alpha-hydroxy acids	-3,26
1369259_at	Dio1	25430	Deiodinase, iodothyronine	-3,26
1387183_at	Crot	83842	Catalyzes the conversion of L-carnitine and octanoyl-coa to L-octanoylcarnitine and coa; plays a role in fatty acid transport	-2,77
1368924_at	Ghr	25235	May play a role in chondrocyte differentiation and positive regulation of longitudinal bond growth	-2,68
1369546_at	Bbox1	64564	Butyrobetaine (gamma)	-2,45
1375247_at	Mgll	29254	Monoglyceride lipase serine hydrolase that converts monoglycerides to fatty acid and glycerol	-2,45
1392994_at	LOC499330	499330	Nicotinamide riboside kinase 1	-2,35
1368536_at	Enpp2	84050	Ectonucleotide pyrophosphatase/phosphodiesterase 2 may regulate oligodendrocyte cell-extracellular matrix adhesive interactions	-2,24
1379885_at	Fmo4	246247	Flavin containing monooxygenase 4	-2,20
1387958_at	Akr1c18	171516	Aldo-keto reductase family 1, catalyses the convariant of progesterone into 20 alpha-dihydroprogesterone	-2,19
1390383_at	Adfp	298199	Perilipin 2 intrinsic lipid storage droplet protein; involved in transfer of lipid between lipofibroblasts and alveolar type II epithelial cells	-2,11
1368272_at	Got1	24401	Glutamic-oxaloacetic transaminase	-2,10
1373686_at	Serpina6	299270	Serpin peptidase inhibitor	-2,10
1398282_at	Kynu	116682	Kynureninase enzyme involved in the biosynthesis of NAD cofactors from tryptophan	-2,08
1386946_at	Cpt1a	25757	Carnitine palmitoyltransferase enzyme that catalyses the transfer of	-2,06

			long chain fatty acids to carnitine	
1388210_at	Acot2	192272	Acyl-coa thioesterase 2 may have a role in lipid metabolism	-2,02
1370566_at	Rdh2	299511	Retinol dehydrogenase 16 a short-chain dehydrogenase/reductase that catalyzes the conversion of retinol to retinal	-2,02
1371363_at	Gpd1	60666	Glycerol-3-phosphate dehydrogenase a mitochondrial glycerol 3-phosphate dehydrogenase	-2,01
1368387_at	Bdh1	117099	3-hydroxybutyrate dehydrogenase	-2,01

Catalytic Activity

1377014_at	RGD1308116	310376	Serine/threonine-protein kinase NIM1	-3,60
1387336_at	Nat8	64570	N-acetyltransferase 8 may act as an N-acetyltransferase; may regulate cell surface adhesion	-3,00
1374883_at	Mtmr7	306490	Myotubularin related protein 7	-2,76
1370938_at	Pdxp	727679	Pyridoxal (pyridoxine, vitamin B6) phosphatase	-2,09
1390607_at	Zdhhc23	363783	Zinc finger, DHHC-type containing 23	-2,03
1368512_a_at	Enpep	64017	Glutamyl aminopeptidase that selectively hydrolyzes acidic amino acid residues from the amino terminus of oligopeptides	-2,01

mRNA 5'-UTR Binding

1390860_at	Igf2bp3	312320	İnsulin-like growth factor 2 mrna binding protein 3	-2,95
1393202_a_at	Igf2bp3	312320	İnsulin-like growth factor 2 mrna binding protein 3	-2,74
1377824_a_at	Igf2bp3	312320	İnsulin-like growth factor 2 mrna binding protein 3	-2,59

Lipid Homeostasis

1388924_at	Angptl4	362850	Angiopoietin-like 4 a circulating protein which causes an increase in plasma very low density lipoprotein	-4,34
1394392_at	Fitm2	311617	Fat storage-inducing transmembrane protein 2	-2,38
1374278_at	Fitm2	311617	Fat storage-inducing transmembrane protein 2	-2,09

Sulphidation

1377672_at	RGD1559960 /// Sult1c2	171072///501084	Similar to Sulfotransferase K1	-2,87
1370943_at	Sult1c2 /// Sult1c2a	171072///316153	Sulfotransferase family	-2,07

Table 7. Genes that were up- or down-regulated relative to the control group and common to both the streptozotocin and resveratrol treated groups.

Common up-regulated genes by streptozotocin and resveratrol				Common down-regulated genes by streptozotocin and resveratrol			
**Probe Set ID**	**Gene Symbol**	**FC Diabetes vs. Control**	**FC K+RSV vs. Control**	**Probe Set ID**	**Gene Symbol**	**FC Diabetes vs. Control**	**FC K+RSV vs. Control**
1387703_a_at	Usp2	15,97	7,41	1383439_at	Npas2	-16,00	-2,90
1381811_at		9,23	4,91	1368168_at	Slc34a2	-7,10	-4,18
1387336_at	Nat8	7,87	3,45	1383956_at	RGD1565709	-5,36	-2,94
1368877_at	Zfp354a	6,46	4,09	1373992_at	MGC108823	-4,57	-3,76
1369467_a_at	Pfkfb1	5,08	2,19	1397004_at		-3,89	-2,45
1387109_at	Por	4,70	2,29	1398698_at		-3,88	-2,35
1383665_at	Lpin2	4,06	2,91	1383486_at		-3,51	-2,81
1372051_at		3,09	2,23	1391481_at		-3,47	-2,90
1382231_at		2,98	2,96	1371412_a_at		-3,40	-3,12
1367577_at	Hspb1	2,92	2,07	1374204_at	Wsb1	-3,35	-3,23
1374903_at	Gcnt2	2,86	2,04	1381973_at	Slc25a30	-2,83	-2,99
1370436_at	Acsm2	2,65	3,52	1368426_at	Crot	-2,69	-3,67
1367847_at	Nupr1	2,50	2,75	1391006_at		-2,64	-2,14
1383013_at	Klf13	2,36	2,28	1377907_at	Snrnp48	-2,63	-2,40
1390731_at		2,11	2,91	1392818_at	Gas5	-2,62	-2,67
				1379815_at		-2,60	-2,12
				1390416_at	Slc25a30	-2,59	-4,44
				1398595_at	Rbm5	-2,59	-3,21
				1371595_at		-2,55	-2,07
				1382978_at		-2,47	-2,04
				1374803_at		-2,45	-2,78
				1386102_at		-2,44	-2,96
				1375933_at	Cldn2	-2,41	-2,27
				1394283_at		-2,40	-4,05
				1394756_at		-2,39	-2,52
				1377934_at		-2,25	-2,46
				1382020_at	Spag9	-2,23	-2,23
				1389986_at		-2,21	-2,40
				1398440_at	Sfrs18	-2,21	-2,48
				1376744_at		-2,20	-2,63
				1381651_at		-2,16	-2,45
				1397999_at		-2,14	-2,17
				1382330_at		-2,12	-2,17
				1369012_at	Inhba	-2,12	-2,30
				1377651_at	Trio	-2,11	-2,03
				1373626_at		-2,10	-2,95
				1381714_at		-2,07	-2,31
				1381732_at		-2,02	-3,21
				1373776_at		-2,00	-2,12

**FC: Fold change value.*

Table 8. Genes that were up- or down-regulated relative to the control group and common to both D+RSV and K+RSV groups.

Common up-regulated genes in D+RSV vs. Control and K+RSV vs. Control				Common down-regulated genes in D+RSV vs. Control and K+RSV vs. Control			
**Probe Set ID**	**Gene Symbol**	**FC D+RSV vs. Control**	**FC K+RSV vs. Control**	**Probe Set ID**	**Gene Symbol**	**FC D+RSV vs. Control**	**FC K+RSV vs. Control**
1387011_at	Lcn2	21,59	35,80	1387985_a_at	Obp3	-54,79	-2,30
1387703_a_at	Usp2	16,27	7,41	1370479_x_at	Obp3	-31,96	-2,32
1381811_at		10,72	4,91	1371412_a_at		-27,25	-3,12
1374070_at	Gpx2	9,22	2,47	1383439_at	Npas2	-12,15	-2,90
1372016_at	Gadd45b	6,67	4,12	1368426_at	Crot	-10,13	-3,67
1368376_at	Nr0b2	6,64	2,65	1390416_at	Slc25a30	-7,28	-4,44
1370436_at	Acsm2	6,18	3,52	1383946_at	Cldn1	-7,14	-9,69
1384243_at	Spsb4	5,79	2,99	1383486_at		-6,76	-2,81
1370445_at	Pla1a	5,35	2,88	1381973_at	Slc25a30	-6,37	-2,99
1391106_at		4,66	2,59	1388229_a_at	Mug1	-6,15	-5,03
1387109_at	Por	4,61	2,29	1377014_at	RGD1308116	-5,97	-3,47
1368007_at	Dmbt1	4,30	2,12	1373992_at	MGC108823	-5,90	-3,76
1372051_at		4,26	2,23	1383956_at	RGD1565709	-4,92	-2,94
1388433_at	Krt19	4,18	2,62	1369577_at	Socs2	-4,15	-2,19
1383665_at	Lpin2	4,16	2,91	1398698_at		-3,77	-2,35
1388901_at	Fkbp5	3,99	2,54	1368168_at	Slc34a2	-3,58	-4,18
1368877_at	Zfp354a	3,96	4,09	1377934_at		-3,57	-2,46
1391791_at	Acer2	3,61	2,11	1391481_at		-3,44	-2,90
1367712_at	Timp1	3,59	2,46	1369012_at	Inhba	-3,34	-2,30
1367847_at	Nupr1	3,46	2,75	1379636_at	Fam82a1	-3,24	-3,73
1394503_at		3,40	2,22	1386002_at		-3,19	-3,38
1367577_at	Hspb1	3,38	2,07	1389612_at		-3,12	-2,57
1369467_a_at	Pfkfb1	3,29	2,19	1378074_at		-3,07	-3,46
1378032_at	Nfkbiz	3,28	2,73	1369737_at	Crem	-3,06	-3,36
1386879_at	Lgals3	3,17	2,04	1368924_at	Ghr	-2,97	-6,54
1367709_at	Cd63	3,13	2,14	1386981_at	Slc16a1	-2,92	-2,03
1387343_at	Cebpd	3,07	2,06	1397004_at		-2,89	-2,45
1369693_a_at	Slc1a2	2,97	2,28	1373626_at		-2,81	-2,95
1371447_at	Plac8	2,79	2,88	1382599_at		-2,70	-3,73
1387125_at	S100a9	2,75	4,89	1394283_at		-2,55	-4,05
1387925_at	Asns	2,72	2,54	1381732_at		-2,54	-3,21
1378015_at	Ccl21b	2,69	2,30	1379815_at		-2,51	-2,12
1387336_at	Nat8	2,62	3,45	1368325_at	Egf	-2,47	-2,44
1377034_at	Serpinb1a	2,62	2,83	1374204_at	Wsb1	-2,44	-3,23
1372691_at	Upp1	2,61	3,60	1398595_at	Rbm5	-2,41	-3,21
1390731_at		2,59	2,91	1398440_at	Sfrs18	-2,39	-2,48
1383401_at	Tes	2,58	2,17	1393285_at		-2,36	-2,03
1378184_at	Cd200r2	2,56	2,27	1375933_at	Cldn2	-2,30	-2,27
1374649_at	Rasgrp2	2,46	2,05	1374752_at	Mdfic	-2,29	-2,40
1368521_at	Napsa	2,35	2,94	1384112_at	Nt5e	-2,28	-3,40
1376390_at	Ms4a11	2,34	2,17	1371595_at		-2,27	-2,07
1386912_at	Pcolce	2,34	2,17	1373776_at		-2,21	-2,12
1370371_a_at	Ceacam1 /// Ceacam10	2,32	2,04	1394756_at		-2,18	-2,52
1372560_at		2,25	2,07	1377907_at	Snrnp48	-2,15	-2,40
1383013_at	Klf13	2,24	2,28	1374574_at	RGD1310552	-2,15	-3,00
1367816_at	Hopx	2,23	2,14	1370908_at	Hdac2	-2,11	-3,12

1387687_at	Igsf6	2,22	2,04	1391006_at		-2,10	-2,14
1388460_at	Capg	2,21	2,66	1397363_at	Pvrl3	-2,08	-3,19
1373889_at	Igsf7 /// LOC363715 /// RGD1561778	2,18	2,55	1373015_at	LOC683844	-2,05	-2,00
1369204_at	Hck	2,13	2,72	1384743_at		-2,03	-2,31
1373025_at	C1qc	2,13	2,40	1383557_at		-2,01	-2,11
1374249_at	RGD1304580	2,12	2,14				
1390336_at	RGD1307569	2,11	2,00				
1379766_at	Sla	2,10	2,28				
1370119_at	Lst1	2,09	2,32				
1388742_at		2,08	2,18				
1388403_at	Idh2	2,07	2,07				
1369029_at	Plscr1	2,06	2,27				
1370023_at	Gja4	2,04	2,02				
1376675_at		2,02	2,46				
1370882_at	RT1-DMb	2,01	2,31				
1389409_at	Tes	2,00	2,62				

**FC: Fold change value.*

Table 9. Fold change values of the genes whose expression profile was modified in all three groups (Diabetes, D+RSV and K+RSV) as compared with control groups

Common up-regulated genes in Diabetes vs. Control, D+RSV vs. Control and K+RSV vs. Control					Common down-regulated genes in Diabetes vs. Control, D+RSV vs. Control and K+RSV vs. Control				
**Probe Set ID**	**Gene Symbol**	**FC Diabetes vs. Control**	**FC D+RSV vs. Control**	**FC K+RSV vs. Control**	**Probe Set ID**	**Gene Symbol**	**FC Diabetes vs. Control**	**FC D+RSV vs. Control**	**FC K+RSV vs. Control**
1387703_a_at	Usp2	15,97	16,27	7,41	1371412_a_at	Nrep	-3,40	-27,25	-3,12
1381811_at		9,23	10,72	4,91	1383439_at	Npas2	-16,00	-12,15	-2,90
1387336_at	Nat8	7,87	2,62	3,45	1368426_at	Crot	-2,69	-10,13	-3,67
1368877_at	Zfp354a	6,46	3,96	4,09	1390416_at	Slc25a30	-2,59	-7,28	-4,44
1369467_a_at	Pfkfb1	5,08	3,29	2,19	1383486_at		-3,51	-6,76	-2,81
1387109_at	Por	4,70	4,61	2,29	1381973_at	Slc25a30	-2,83	-6,37	-2,99
1383665_at	Lpin2	4,06	4,16	2,91	1373992_at	MGC108823	-4,57	-5,90	-3,76
1372051_at		3,09	4,26	2,23	1383956_at	RGD1565709	-5,36	-4,92	-2,94
1367577_at	Hspb1	2,92	3,38	2,07	1398698_at		-3,88	-3,77	-2,35
1370436_at	Acsm2	2,65	6,18	3,52	1368168_at	Slc34a2	-7,10	-3,58	-4,18
1367847_at	Nupr1	2,50	3,46	2,75	1377934_at		-2,25	-3,57	-2,46
1383013_at	Klf13	2,36	2,24	2,28	1391481_at		-3,47	-3,44	-2,90
1390731_at		2,11	2,59	2,91	1369012_at	Inhba	-2,12	-3,34	-2,30
					1397004_at		-3,89	-2,89	-2,45
					1373626_at		-2,10	-2,81	-2,95
					1394283_at		-2,40	-2,55	-4,05
					1381732_at		-2,02	-2,54	-3,21
					1379815_at		-2,60	-2,51	-2,12
					1374204_at	Wsb1	-3,35	-2,44	-3,23
					1398595_at	Rbm5	-2,59	-2,41	-3,21
					1398440_at	Sfrs18	-2,21	-2,39	-2,48
					1375933_at	Cldn2	-2,41	-2,30	-2,27
					1371595_at		-2,55	-2,27	-2,07
					1373776_at		-2,00	-2,21	-2,12
					1394756_at		-2,39	-2,18	-2,52
					1377907_at	Snrnp48	-2,63	-2,15	-2,40
					1391006_at		-2,64	-2,10	-2,14

**FC: Fold change value.*
